# Overexpression of vacuolar H^+^-pyrophosphatase from a recretohalophyte *Reaumuria trigyna* enhances vegetative growth and salt tolerance in transgenic *Arabidopsis thaliana*

**DOI:** 10.3389/fpls.2024.1435799

**Published:** 2024-11-13

**Authors:** Ningning Li, Yuzhu Cui, Zijian Zhang, Shuai Wang, Yaqing Sun, Shaoying Zhang, Guolong Li

**Affiliations:** College of Agronomy, Inner Mongolia Agricultural University, Hohhot, China

**Keywords:** H^+^ -pyrophosphatase, vegetative growth, transgenic *Arabidopsis*, *Reaumuria trigyna*, salt tolerance

## Abstract

*Reaumuria trigyna*, a wild and endangered salt-secreting small shrub, is distributed in arid and semi-arid areas of Inner Mongolia, China. An H^+^-pyrophosphatase gene (*RtVP1*) was isolated from *R*. *trigyna* according to transcriptomic data, which encoded a plasma membrane and tonoplast-localized protein. *RtVP1* was quickly upregulated by NaCl and exogenous abscisic acid treatment and rescued the sucrose deficiency sensitive phenotype of the *AtVP1* mutant (*avp1*). Transgenic *Arabidopsis* overexpressing *RtVP1* exhibited a higher leaf area, plant height, fresh weight, root length, and soluble carbohydrate accumulation compared to the wild type (WT) under normal conditions. *RtVP1* overexpression increased the seed germination rate and decreased the reduction rate of fresh weight, root length, and chlorophyll content in transgenic plants under salt stress. Catalase enzyme activity, proline content, relative water content, and soluble sugar content were significantly increased in transgenic *Arabidopsis* under salt stresses, but the malondialdehyde content was dramatically decreased. More K^+^ and less Na^+^ were accumulated in transgenic *Arabidopsis* leaves, resulting in a relatively lower Na^+^/K^+^ ratio. In transgenic *Arabidopsis* roots, K^+^ was unchanged, but Na^+^ and the Na^+^/K^+^ ratios were reduced compared to those in WT. More Na^+^ and K^+^ were accumulated in the intracellular of transgenic yeast, and the Na^+^/K^+^ ratio was significantly reduced compared to the control. These results showed that *R*. *trigyna RtVP1* promotes the vegetative growth of plants, mainly by regulating carbohydrate metabolism, and confers salt tolerance in transgenic *Arabidopsis* by maintaining Na^+^/K^+^ homeostasis and enhancing the antioxidant and osmotic regulatory capacity. These results indicated that *RtVP1* can serve as an important candidate gene for genetic improvement of crop yield and salt tolerance.

## Introduction

Plant vacuolar-type H^+^-pyrophosphatases (V-H^+^-PPases, EC 3.6.1.1) can utilize the energy released by the hydrolysis of inorganic pyrophosphate (PPi) to drive H^+^ transmembrane transport and is found in almost all plants in nature (Maeshima, 2000). Prototypical plant H^+^-PPases exhibit high conservation in different plant species and are classified into two types based on their sensitivity to K^+^: K^+^-activated and K^+^-insensitive (Drozdowicz et al., 2000; Drozdowicz and Rea, 2001). Thus far, vacuolar H^+^-PPase genes have been widely characterized in various plant species, particularly with a focus on their association with the acidification of vacuoles and the alkalization of the cytoplasm (Kriegel et al., 2015), regulation of auxin distribution and organ development (Li et al., 2005; Cheng et al., 2018), enhancement of root acidification (Yang et al., 2015; Kim et al., 2020), improvement of nutrient uptake and use efficiencies (Paez-Valencia et al., 2013; Li et al., 2014; Fukuda et al., 2016), photoassimilate phloem loading and long-distance transport (Paez-Valencia et al., 2013; Pizzio et al., 2015; Khadilkar et al., 2016), and regulation of seedling development and PPi homeostasis (Fukuda et al., 2020; Tojo et al., 2023). In addition, overexpression of vacuolar H^+^-PPases in numerous plant species increases root and shoot biomass accumulation (Graus et al., 2018; Kim et al., 2020), which also improves salt tolerance and drought resistance (Schilling et al., 2014; Lv et al., 2015; Zhang et al., 2016; Yu et al., 2017; Cui et al., 2019; Toranj et al., 2020; Fu et al., 2022).

To date, it has been demonstrated that vacuolar H^+^-PPases can improve transgenic plants’ vegetative growth and enhance plant biomass accumulation. For example, *AtVP1*-overexpressing *Arabidopsis* exhibits more leaf numbers and larger leaf areas than wild type (WT) because of increased cell numbers and enhanced root growth and dry weight, which may be due caused by the increased H^+^-ATPase and PIN1 abundance, thus regulating the indole acetic acid (IAA) flux through cells (Li et al., 2005). Gonzalez et al. (2010) reported that *AtVP1* overexpression enhanced the leaf area, length, and width and petiole length of transgenic *Arabidopsis*; increased the cell numbers but no change in cell areas; and significantly increased the IAA, salicylic acid, abscisic acid (ABA), and gibberellic acid contents in transgenic plants. Transgenic cotton overexpressing *ThVP* from *T*. *halophila* exhibited a higher shoot and root fresh weight and maintained higher net photosynthesis, intercellular CO_2_ concentration, stomatal conductance, and soluble sugar content than WT plants. Furthermore, Pizzio et al. (2015) reported that *AtVP1* was located on the plasma membrane of the sieve element–companion cell complexes, which act as a PPi synthase using proton motive force to maintain PPi homeostasis and that both ubiquitous and phloem-specific *AtVP1* overexpression increased shoot fresh weight, production and transport of photosynthetic products, the expression of sugar induced root ion transport genes, and rhizosphere acidification. C_14_-labeling experiments showed that overexpression of *AtVP1* increased photosynthesis and sucrose metabolism and improved phloem loading and transport to sink organs, indicating that AtVP1 plays a role as a PPi synthase in phloem, thereby promoting stem biomass accumulation (Khadilkar et al., 2016). These results suggest that vacuolar H^+^-PPases can enhance the vegetative growth of plants and promote biomass accumulation by regulating various metabolic pathways, such as plant hormone distribution, photosynthesis efficiency, and sucrose metabolism, and by promoting the transport of photosynthetic products from source to sink.

Vacuolar H^+^-PPases enhance salt tolerance in plants by regulating Na^+^/K^+^ homeostasis, improving the capacity for antioxidant regulation, and osmotic adjustment. For example, transgenic poplar plants expressing *PtVP1*.*1* exhibited a higher plant height, shoot and root biomass, and survival rate and a higher K^+^ and lower Na^+^ accumulation in leaves and increased the Na^+^ flux and H^+^ influx in root cells compared to WT plants under salt stress, suggesting that *PtVP1*.*1* can maintain the Na^+^/K^+^ homeostasis to alleviate the Na^+^ toxic in leaf and thus enhance the salt tolerance of transgenic poplar (Yang et al., 2015). Transgenic tobacco expressing *IlVP* from *Iris lactea* exhibited vigorous growth a higher Na^+^ and K^+^ contents in leaves, stems, and roots and maintained a higher relative membrane permeability and relative water content (RWC) compared to WT under salt stress, implying that *IlVP* enhances plant salt tolerance by maintaining the Na^+^/K^+^ homeostasis and improving the capacity for osmotic regulation (Meng et al., 2017). Leaves of transgenic finger millet overexpressing *SbVPPase* (*Sorghum bicolor*) had a higher Na^+^, K^+^, and proline content and superoxide dismutase (SOD), ascorbate peroxidase, catalase (CAT), glutathione peroxidase, and glutathione reductase and a lower malondialdehyde (MDA) content compared to WT plants under salt stress, indicating that SbVPPase can maintain the ion homeostatic balance and improve the scavenging efficiency of reactive oxygen species (ROS) and osmotic regulation in transgenic plants, consequently alleviating salt stress (Anjaneyulu et al., 2014). Similarly, overexpression of *ZmVP1* from *Zoysia matrella* and *AnVP1* from *Ammopiptanthus nanus* increased the Na^+^, K^+^, and proline content in transgenic plants under salt stress and decreased the MDA content and relative electrical conductivity, suggesting that *ZmVP1* and *AnVP1* enhance antioxidant regulation and osmotic adjustment to improve the salt tolerance of transgenic plants (Chen et al., 2015; Yu et al., 2017). Overexpression of the *OsVP1* gene significantly increased the activity of vacuolar pyrophosphatase and promoted ion absorption and the expression of vacuolar cation antiporter gene, thereby improving the salt tolerance of transgenic plants and promoting root growth (Kim et al., 2020). However, previous studies on the function of H^+^-PPase genes have mostly been based on model plants and crops, and the H^+^-PPase function of archaic wild recretohalophytes has not been sufficient.

*Reaumuria trigyna* (Tamaricaceae), an ancient dicotyledonous small shrub originating from the Tethys Ocean, is mainly distributed in arid and semi-arid plateau areas of Inner Mongolia (106°27′E–111°28′E, 39°13′N–40°52′N, elevation 1,500–2,100 m) (Li, 1990; Yang et al., 2002; Zhao, 2006). It is a typical recretohalophyte that can survive in habitats with a salinity of 0.7%, which may be attributed to its unique morphological and physiological adaptability, including salt glands on the surface of leaves and stems, acicular succulent leaves, and well-developed roots (Xue and Wang, 2008). Xue et al. (2012a, b) found that the content of proline, soluble sugar, and free amino acid were significantly increased in *R. trigyna* under salt stress, and the activities of SOD and peroxidase (POD) were also significantly enhanced. Dang et al. (2013) demonstrated that multiple genes related to ion transport system, ROS scavenging system, and osmotic regulation system were significantly upregulated after salt stress, suggesting that *R. trigyna* can improve salt tolerance by regulating ion homeostasis balance, antioxidant regulation, and osmotic regulation. Many ion transporters involved in maintaining Na^+^/K^+^ homeostasis can enhance salt tolerance, and these ion transporter genes may also play roles in other salt-tolerance mechanisms, such as ROS scavenging and osmoregulation. Previously, we isolated two transporters from *R*. *trigyna*, a vacuolar Na^+^/H^+^ antiporter gene (*RtNHX1*) and a high-affinity potassium transporter gene (*RtHKT1*). *RtNHX1* can sequester Na^+^ and K^+^ in the vacuole, maintain a relatively low Na^+^/K^+^ ratio in leaves, promote the accumulation of osmotic adjustment substances, improve the activity of antioxidant enzymes, and then enhance the salt tolerance of transgenic Arabidopsis (Li et al., 2017). *RtHKT1* mediates K^+^ uptake and prevents Na^+^ transport to leaves in response to salt stress and improves the osmoregulation ability and ROS scavenging efficiency, thereby improving salt tolerance of transgenic plants (Li et al., 2019). In present study, a vacuolar H^+^-pyrophosphatase gene (*RtVP*1) was isolated and its function was systematically investigated to gain insight into the molecular regulation mechanism of vegetative growth and salt tolerance. This study will provide a candidate gene for genetic improvement the vegetative growth and salt tolerance in crops and herbage.

## Materials and methods

### Plant materials

Seeds of *R*. *trigyna* were obtained from the arid and semi-arid regions of Inner Mongolia, China. The seeds were sterilized with 10% sodium hypochlorite for 15 min, then sown on Murashige and Skoog (MS) solid medium (Murashige and Skoog, 1962) for 72 h in the dark for seed germination, and then placed in 70% relative humidity, 25°C, and 16-h light/8-h dark cycle environment for growth. Seedlings grown to 10 cm were transferred to half-strength Hoagland’s liquid medium for 4 weeks, and the medium was changed every 48 h. Seedlings of similar size were treated with 100 mM, 200 mM, 300 mM, 400 mM, and 500 mM NaCl. To prevent osmotic shock, the NaCl concentration was increased by 100 mM per step every 8 h to reach a final concentration. Leaves were collected after 24 h at each increase in NaCl concentration and after the seedlings were treated in 200 mM NaCl and 100 µM ABA for 0, 1, 3, 6, 12, and 24 h. Samples were rapidly frozen in liquid nitrogen and stored at −80°C.

*Arabidopsis thaliana* seeds of Columbia-0 (Col-0) and *avp1* (GK-377B04-026822) genotypes were purchased from GABI-KAT (http://www.gabi-kat.de/). For the acquisition and culture of *Arabidopsis* seedlings, see the previous description Li et al. (2017).

### Isolation of the *RtVP1* gene

To isolate *RtVP1* cDNA from *R*. *trigyna*, total RNA was obtained using a Plant RNA Extraction Kit (TaKaRa Bio Inc., Otsu, Shiga, Japan), and the 5′-rapid amplification of cDNA ends (RACE) PCR was performed using the SMART RACE cDNA Amplification Kit (Clontech, Mountain View, CA, USA). The primers 5′RACE-R1 and 5′RACE-R2 were designed according to the *RtVP1* cDNA fragment from the transcriptome database (Dang et al., 2013). The full-length *RtVP1* cDNA was obtained using the primer sets RtVP1-Full-F and RtVP1-Full-R. PCR reaction conditions were as follows: 94°C for 30 s, 53°C for 45 s, and 72°C for 2 min for 35 cycles. The PCR products were cloned into the pEASY-T1 vector for DNA sequencing by BGI (Beijing, China).

### Phylogenetic analysis

Phylogenetic analysis was conducted using amino acid sequences of 19 VPs from 13 species, such as *Populus trichocarpa*, *Arabidopsis thaliana*, *Brassica napus*, *Camelina sativa*, *Prunus persica*, *Salicornia europaea*, *Nicotiana tabacum*, *Gossypium hirsutum*, *Glycine max*, *Prunus mume*, *Beta vulgaris*, and *Sesamum indicum*. The phylogenetic tree was performed in MEGA5 using the neighbor-joining method.

### Subcellular localization analysis

To investigate the subcellular localization of the RtVP1 protein, the open reading frame (ORF; excluding the termination codon) of *RtVP1* was inserted into the pUC18-sGFP vector to produce pUC18-35S-RtVP1::sGFP. RtVP1-*Sal*I-F and RtVP1-*Sal*I-R (**Supplementary Table S1**) were used to amplify *RtVP1* ORF, and the pUC18-sGFP vector was digested by *Sal*I. The recombinant vector was transformed into onion epidermal cells using a *Biolistic PDS-1000/HE* particle delivery system (Bio-Rad, Hercules, CA, USA) as described by Huang et al. (2020). Green fluorescence was monitored under a fluorescence microscope (*ECLIPSE 80i*, *Nikon*, Tokyo, Japan).

### Real-time PCR and RT-PCR analysis

To determine the *RtVP1* expression pattern, real-time PCR was performed with *RtVP1*-RT-F and *RtVP1*-RT-R (**Supplementary Table S1**), and the *RtActin* gene was used as an internal control. The following stress-related genes were also monitored by real-time PCR: *AtSOD1* (NM_100757), *AtPOD1* (NM_112583.2), *AtCAT1* (AT1G20630), *AtP5CS1-2* (AT2G39800 and AT1G55610), *AtSOS1* (AT2G01980), *AtNHX1* (NM_122597.2), *AtHAK5* (At4g13420), *AtKUP8* (At5g14880), *AtSUS1-6* (At5g20830, At5g49190, At4g02280, At3g43190, At5g37180, and At1g73370), *AtAHA1-4* (At2g18960, At4g30190, At5g57350, and At3g47950), *AtPFKα1-2* (At1g20950 and At1g7655), *AtPFKβ1-2* (At1g12000 and At4g04040), *AtUGP1-2* (At3g03250 and At5g17310), *AtSWEET11-12* (At3g48740 and At5g23660), and *AtCIN1-2* (At1g35580 and At4g09510), and *AtActin8* (NM_103814.4) gene was used as a reference gene (Guo et al., 2015). The qPCR was performed with the a *TransStart* Tip Green qPCR SuperMix Kit (TransGen Biotech) in a LightCycler^®^ 480II platform (Roche, Hilden, Switzerland). The reaction conditions were as follows: 94°C for 30 s, followed by 48 cycles of 94°C for 5 s, 58°C for 15 s, and 72°C for 10 s. The relative expression levels of target genes were analyzed by 2^−△△CT^ method as described by Livak and Schmittgen (2001). For the RT-PCR, the primers RtVP1-SP-F and RtVP1-SP-R (**Supplementary Table S1**) were used to amplify *RtVP1*, and *AtActin* 2 gene was used as a reference gene (Lin et al., 2008), The reaction conditions were as follows: 35 cycles of 94°C for 30 s, 56°C for 30 s, and 72°C for 1 min. All experiments were performed with three biological replicates and three technical replicates.

### Genetic transformation of *Arabidopsis*

The pEASY-T1-*RtVP1* vector and pBI101 vector were digested by *Xba*I and *Sac*I simultaneously, and then, *RtVP1* ORF was inserted into the pBI101 plasmid to construct the pBI101-RtVP1 plasmid. The recombinant vector was introduced into *Agrobacterium tumefaciens* (LBA4404) and genetically transformed into *A*. *thaliana* (WT and *avp1*) through simplified in-plant infiltration method described by Kim et al. (1999). The kanamycin (40 mg L^–1^) was used for screening transgenic line. The genomic PCR was conducted using the NPTII-F and NPTII-R (**Supplementary Table S1**) for validating transgenic lines. Transgenic homozygous lines were obtained through self-pollination of kanamycin resistant plants. All analyses were carried out with the T3 transgenic plants of each homozygous line.

### H^+^-PPase activity measurements

Preparation of leaf microsomal fractions and detection of H^+^-PPase activity referred to the previous description by Tang et al. (2012).

### Measurement of the physical and physiological parameters of transgenic *Arabidopsis*

The LI-3000C Portable Area Meter was used to measure the area, length, and width of the fifth leaf of *A. thaliana*. The physiological parameters of all lines, such as glucose, fructose, sucrose, soluble sugars, starch, amino acids, protein, and inorganic phosphorus content, were measured using the corresponding assay kits (Comin Biotechnology Co. Ltd, Suzhou). The SPAD-502 was used to measure the chlorophyll content. The RWC was calculated as follows: (fresh weight − dry weight)/(turgescent weight − dry weight). The activities of POD and CAT and the content of MDA and proline were measured using the corresponding assay kits from the Jiancheng Bioengineering Institute (Nanjing).

### Salt tolerance analysis of transgenic *Arabidopsis*

The seed germination rates were monitored at 7 days after planting on MS medium containing 0 mM and 100 mM NaCl. Seven-day-old seedlings were moved to MS medium containing zero and 100 mM NaCl, and the seedling fresh weight and root length were monitored at 7 days after transfer onto vertically placed plates. Thirty-day-old soil-grown plants were gradually treated with 25 mM NaCl salt solution twice daily until a final concentration of 100 mM and 200 mM, and the physiological parameters, such as mature fresh weight, chlorophyll content, POD, CAT, MDA, RWC, proline content, and soluble sugar content, were measured after 10 days of 100 mM and 200 mM NaCl salt stress.

### Determination of Na^+^ and K^+^ content

For Na^+^ and K^+^ content determination, the roots and leaves were dried to a constant mass at 80°C and ground into pieces. After digestion with 10% HNO_3_, the fragments were removed by centrifugation. The supernatant was analyzed by inductively coupled plasma optical emission spectroscopy. Three biological replicates for each sample were performed for this experiment.

### Yeast transformation and cation tolerance test

The pYES2-RtVP1 vector was constructed and transformed into AXT3 yeast (Quintero et al., 2002). The genetic transformation method of yeast referred to the previous description by Elble (1992). Yeast cultures (5 µL) with optical density at 600 nm for 0.8 or 10-fold serial dilutions were dropped onto dropout plates containing 100 mM NaCl, 1 M KCl, or 20 mM LiCl and grown at 30°C for 4 days. The detection of Na^+^ and K^+^ content in cytoplasmic, vacuolar, and intracellular referred to the previous description by Li et al. (2017).

### Statistical analysis

The data analysis was conducted using SPSS 18.0 software (Statistical Product and Service Solutions). Duncan’s multiple range test (MRT) was used to evaluate statistical differences. When the probability (*P*) value of < 0.05, there was a significant difference between the means.

## Results

### Isolation and sequence analysis of the *RtVP1* gene

A 2,292-bp *RtVP1* ORF encoding a protein of 763 amino acids with a calculated molecular mass of 79.8 kDa was cloned by RACE-PCR and RT-PCR. The deduced RtVP1 amino acid sequence shared a high consistent with H^+^-pyrophosphatases from other species, such as *Glycine max* (90%), *Nicotiana tabacum* (90%), *Populus trichocarpa* (90%), *Prunus persica* (90%), *Gossypium hirsutum* (89%), and *Arabidopsis thaliana* (88%). Similar to H^+^-pyrophosphatases from these plants, RtVP1 contained five highly conserved motifs and 14 putative transmembrane domains as described by Drozdowicz and Rea (2001) and Maeshima (2000) (**Supplementary Figure S1**). Phylogenetic analysis revealed that RtVP1 was belonged to K^+^-sensitive type vacuolar H^+^-PPases, which were clustered with H^+^-PPase from Rosaceae, such as *P*. *persica* and *P*. *mume* (**Supplementary Figure S2**). These results indicate that RtVP1 might act as a vacuolar H^+^-PPase in *R*. *trigyna*, and its characteristics were similar to that of *P*. *persica* and *P*. *mume*.

### Subcellular localization of RtVP1::sGFP fusion protein

**Figure 1:**
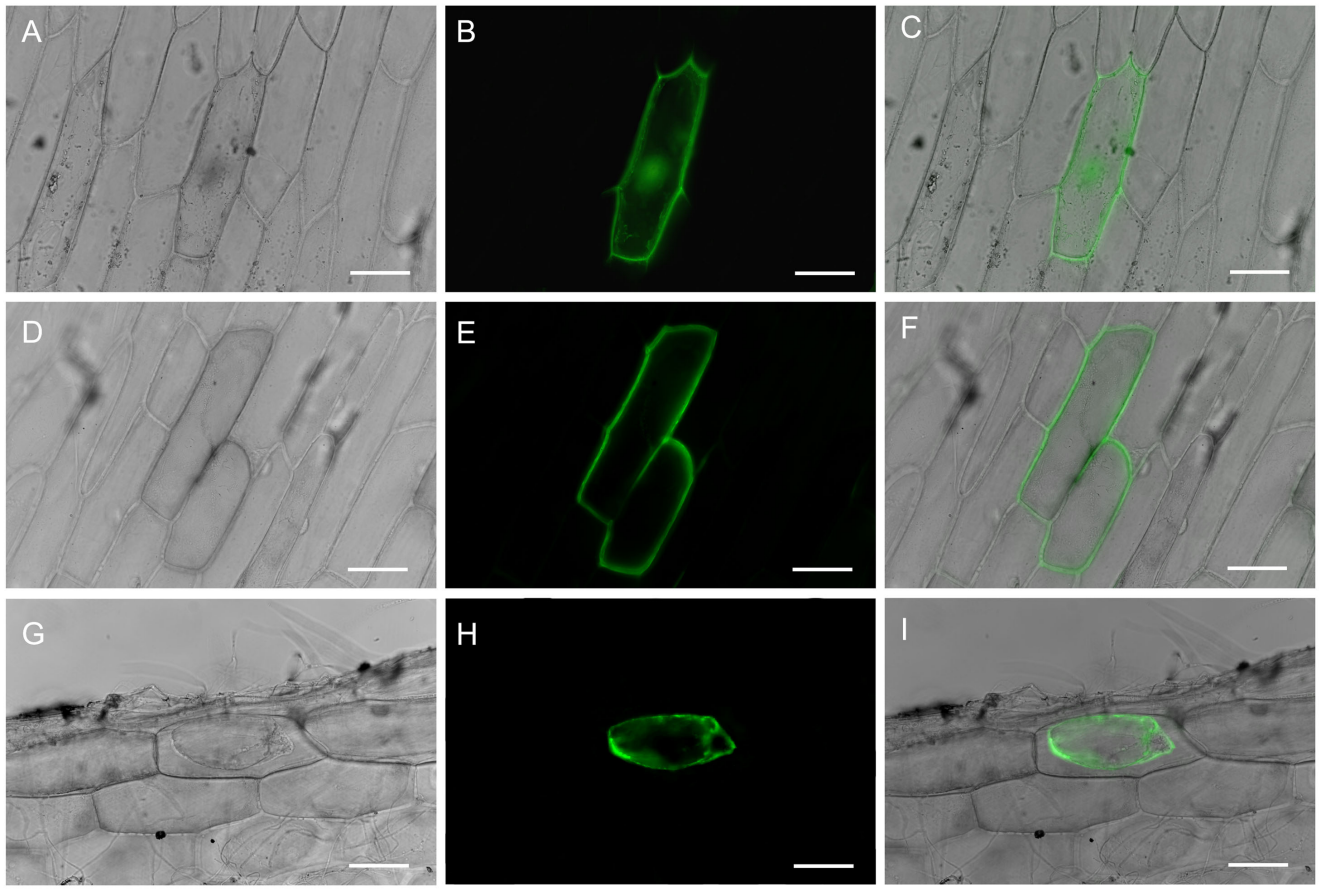
Subcellular localization of the RtVP1 protein. Transient expression of GFP was detected in the nuclei and cytoplasm of onion epidermal cells transformed with the pUC18-sGFP vector [**(A)** bright-field image, **(B)** fluorescence image, and **(C)** merged image], whereas the expression of RtVP1::sGFP fusion protein was detected in the plasma membranes and vacuole membranes of onion epidermal cells before [**(D)** bright-field image, **(E)** fluorescence image, **(F)** and merged image] or after treatment with sucrose (0.3 g/mL) [**(G)** bright-field image, **(H)** fluorescence image, and **(I)** merged image]. The scale bar represents 100 µm.

To determine RtVP1 location, RtVP1::sGFP vector was transiently transformed into onion epidermal cells by particle bombardment. The green fluorescence was found in the plasma membrane and tonoplast of cell transformed with RtVP1::sGFP vector (**Figures 1D–I**), whereas green fluorescence derived from control cells expressing the pUC18-sGFP vector was distributed in the nucleus and cytoplasm (**Figures 1A–C**), indicating that RtVP1 is a plasma membrane and tonoplast-localized protein.

### Expression pattern of *RtVP1* under abiotic stress

**Figure 2:**
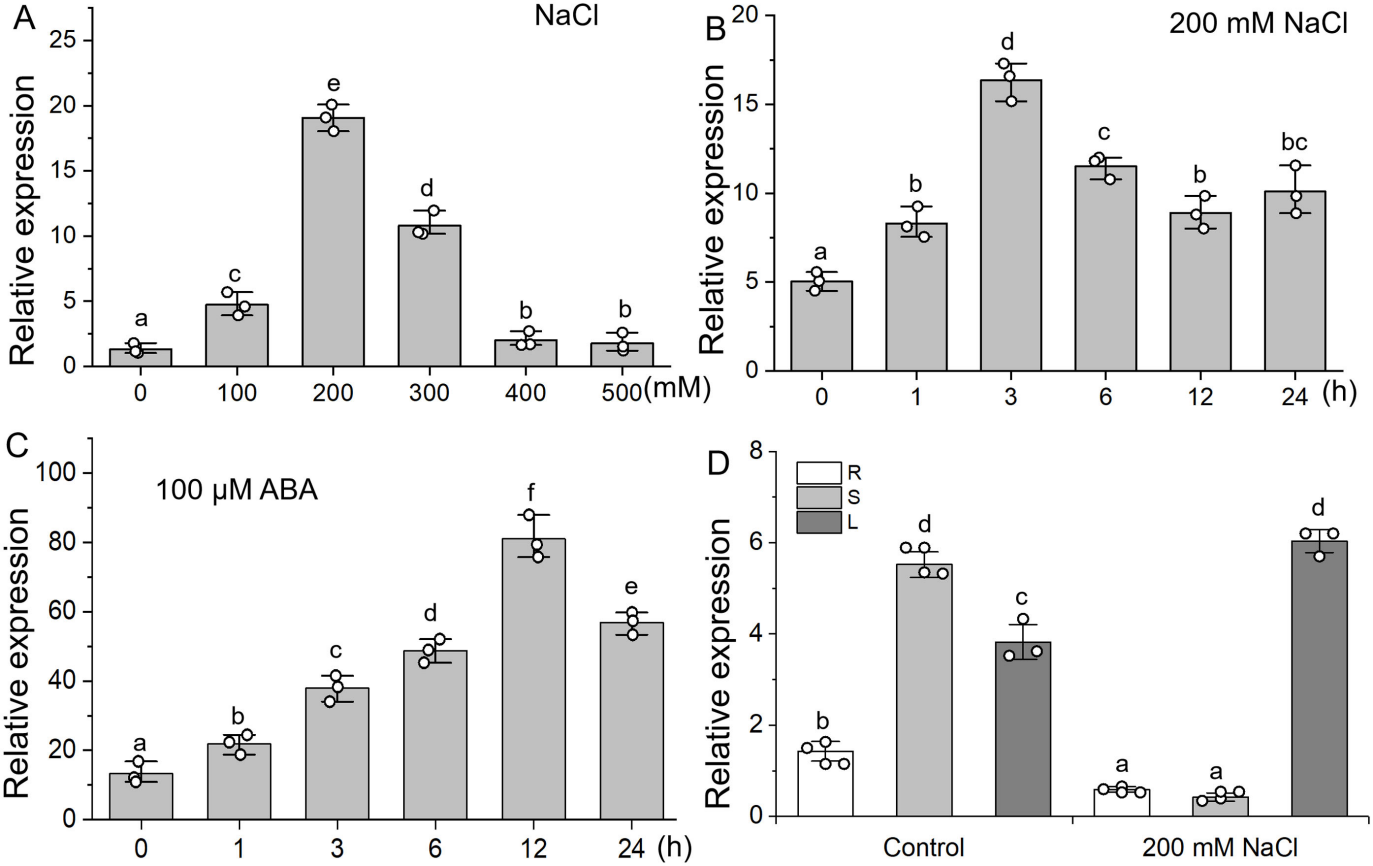
Expression pattern of the *RtVP1* gene under salt stress. **(A)** Relative expression of *RtVP1* under various concentrations of NaCl for 24 h at various times (0, 1, 3, 6, 12, and 24 h) with 200 mM NaCl **(B)** and 100 µM ABA **(C)**. **(D)** Relative expression of *RtVP1* in leaves (L), stems (S), and roots (R) of 40-day-old *Reaumuria trigyna* seedlings under normal conditions and 200 mM NaCl treatment. The *RtActin* gene was used as an internal control. Data are expressed as means ± SE. Values with the same letter are not significantly different [*P* < 0.05, Duncan’s multiple range test (MRT)].

Real-time PCR was used to examine the expression of *RtVP1* in response to salt stress. The expression of *RtVP1* was induced by NaCl and exogenous ABA treatment (**Figures 2A–C**). With the increase of NaCl concentration, *RtVP1* expression increased first and then decreased and was the highest under 200 mM NaCl (**Figure 2A**). The expression of *RtVP1* was reached the peak at 3 h in response to 200 mM NaCl and at 6 h in response to 100 µM ABA (**Figures 2B, C**). *RtVP1* expression in stems was significantly higher than that in roots and leaves under normal conditions, but the expression of *RtVP1* in leaves increased sharply, which was significantly higher than that in roots and stems under salt stress (**Figure 2D**).

### Verification of the *RtVP1* gene in transgenic *Arabidopsis*

**Figure 3:**
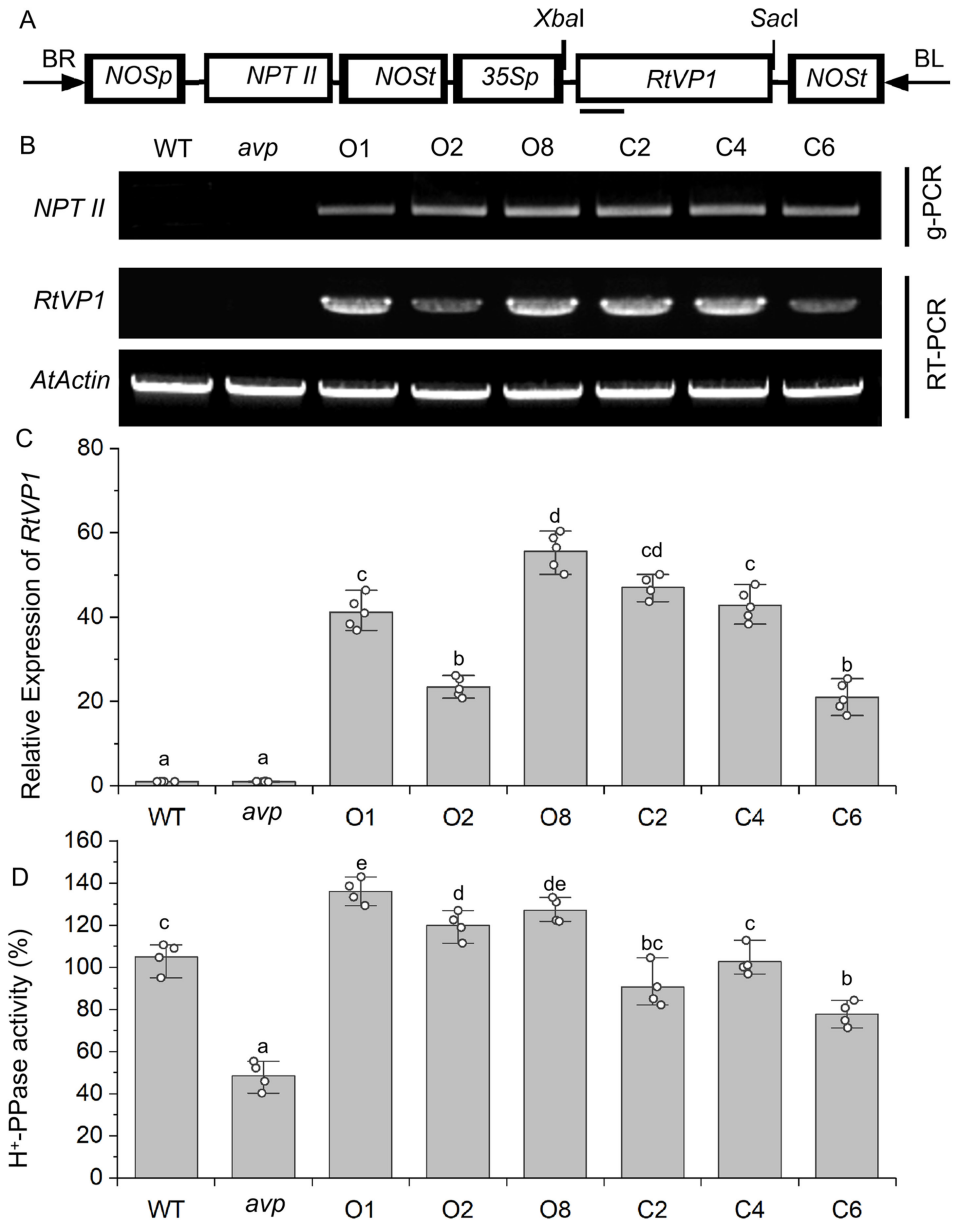
Molecular identification of *RtVP1*-transformed *Arabidopsis thaliana* lines. **(A)** T-DNA region of the vector pBI101-*RtVP1* used to produce transgenic *RtVP1* plants. *35Sp*, cauliflower mosaic virus 35S promoter; *NOSp*, nopaline synthase promoter; *NOSt*, nopaline synthase terminator; *NTP II*, neomycin phosphotransferase II gene; *RB*, right border; *LB*, left border. **(B)** Genomic PCR and RT-PCR analysis to confirm insertion of *RtVP1* into the genome and expression of *RtVP1* in transgenic lines, respectively. The *AtActin* gene was used as an internal control. **(C)** The relative expression levels of *RtVP1* were further quantified in the WT, *avp1*, and transgenic lines using real-time RT-PCR. **(D)** H^+^-PPase hydrolytic activity of WT, *avp1*, and transgenic *Arabidopsis*. O1, O2, and O8 represent transgenic lines overexpressing *RtVP1* in WT, and C2, C4, and C6 represent transgenic lines overexpressing *RtVP1* in *avp1* mutant. Values with the same letter are not significantly different [*P* < 0.05, Duncan’s multiple range test (MRT)].

The pBI101-*RtVP1* vector was introduced into WT and transferred-DNA (T-DNA) insertion *avp1* mutant plants by *Agrobacterium*-mediated genetic transformation (**Figure 3A**). RT-PCR was used to validate the three randomly selected homozygous lines overexpressing *RtVP1* in WT (O1, O2, and O8) and *avp1* mutant (C2, C4, and C6) (**Figure 3B**). Transgenic plants showed higher *RtVP1* expression and H^+^-PPase activity than WT and *avp1* mutants (**Figures 3C, D**).

### RtVP1 protein is a functional homolog of AtVP1

**Figure 4:**
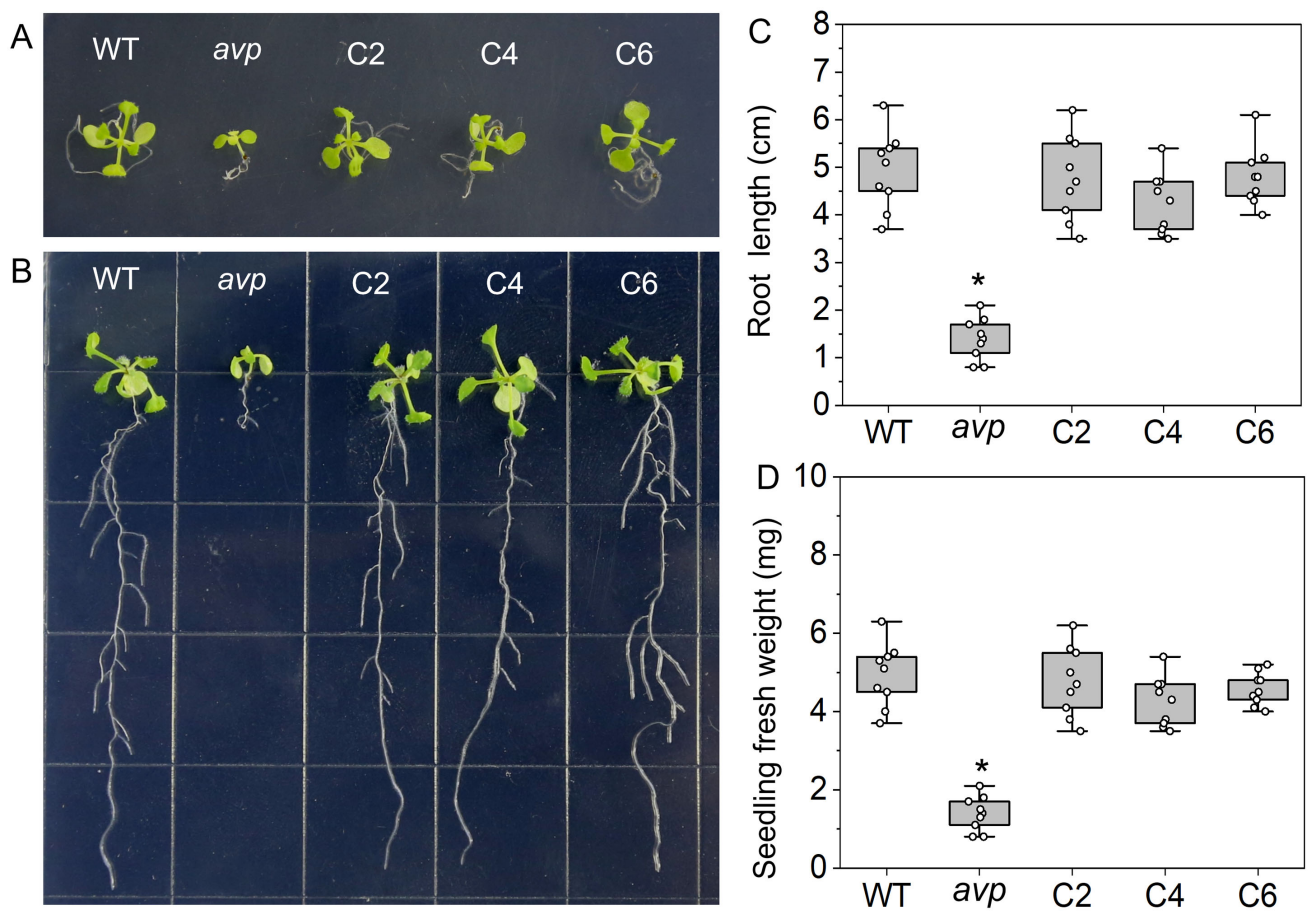
Functional complementation of the *Arabidopsis avp1* mutant by *RtVP1*. Shoot **(A)** and root **(B)** growth of 3-week-old WT, *avp1*, and *avp1* mutant–overexpressing *RtVP1* (C2, C4, and C6) on MS medium without sucrose. Photographs were taken on the 16 days after seed germination. The root length **(C)** and fresh weight **(D)** of seedlings were measured at the end of treatment. Data are expressed as means ± SE. An asterisk (*) indicates a significant difference (*P* < 0.05, Duncan’s multiple range test: *avp1* versus WT and transgenic plants).

**Figure 5:**
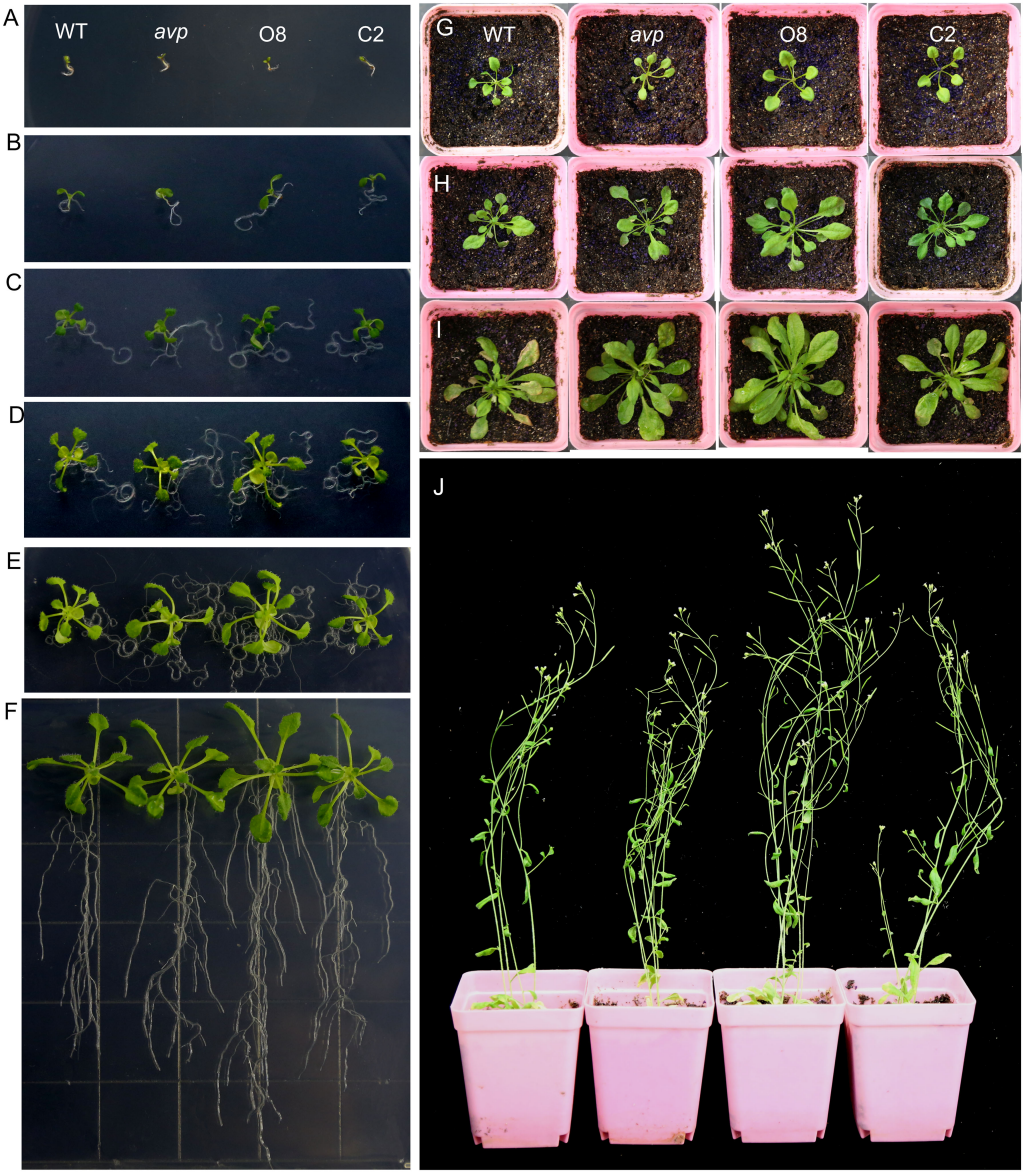
Effects of *RtVP1* overexpression on transgenic *Arabidopsis thaliana*. **(A–E)** WT, *avp1*, and transgenic plants (O8 and C2) cultivated on MS medium at 3 **(A)**, 5 **(B)**, 7 **(C)**, 10 **(D)**, and 14 days **(E)** after sowing. **(F)** Plants of WT, *avp1*, and transgenic plants cultivated for 2 weeks on vertical plates showing the difference in root growth and development. **(G)** Three-week-old WT plants overexpressing *RtVP1* (O8) showed significantly more growth than WT *avp1* and C2 plants. **(H, I)** Four-week-old **(H)** and 5-week-old **(I)** O8 plants showed a faster growth rate than WT *avp1* and C2 plants. **(J)** WT plants overexpressing *RtVP1* (O8) were considerably taller than WT *avp1* and C2 plants at 6 weeks after sowing.

**Figure 6:**
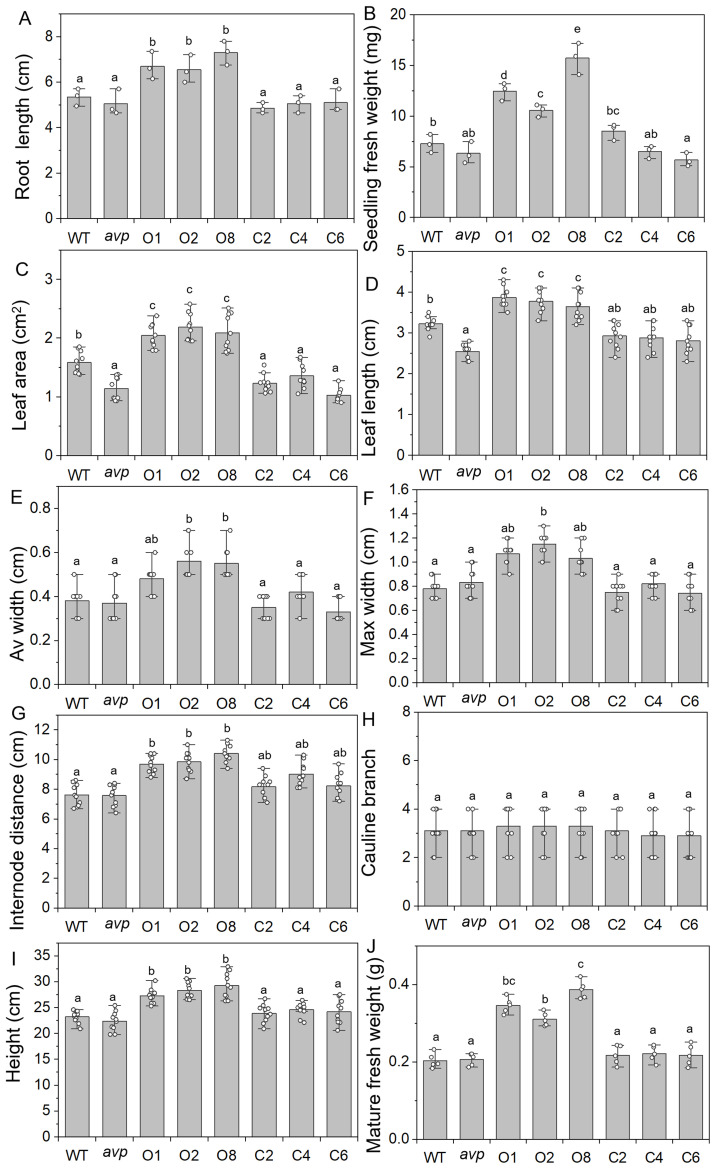
Morphology analysis of WT, *avp1*, and transgenic lines *overexpressing RtVP1* in WT (O1, O2, and O8) and *avp1* mutant (C2, C4, and C6). **(A, B)** Root length **(A)** and seedling fresh weight **(B)** were detected in 2-week-old plants. **(C–J)** The leaf area **(C)**, length **(D)**, average width **(E)**, and max width **(F)** of the fifth rosette, internode distances **(G)**, the number of cauline branches **(H)**, plant height **(I)**, and mature fresh weight **(J)** were measured in 40-day-old plants. Data are expressed as means ± SE. Values with the same letter are not significantly different [*P* < 0.05, Duncan’s multiple range test (MRT)].

To further investigate the function of *RtVP1*, a complementary assay was performed by transforming *RtVP1* into the *Arabidopsis* mutant containing the T-DNA insertion in the exon of *AtVP1*. Previous reports have shown that *Arabidopsis* mutants of *AtVP1* display a significant growth reduction in the absence of sucrose medium but no growth impairment under sucrose supplementation (Pizzio et al., 2015; Yang et al., 2015). As expected, the growth and development of the *avp1* mutant were more adversely affected in the absence of sucrose (**Figures 4A, B**). The *avp1* mutant exhibited lower fresh weight and root length than WT and *RtVP1*-overexpressing *avp1* plants, whereas no obvious differences were noted between WT and *RtVP1*-overexpressing *avp1* plants (**Figures 4C, D**). The growth phenotype of *avp1* mutant plants was indistinguishable from that of *avp1* mutant–overexpressing *RtVP1* plants and WT plants in presence of sucrose medium (**Figures 5E, F**); no apparent differences were noted in root length or fresh weight (**Figures 6A, B**). These results suggest that *RtVP1* overexpression in the *avp1* mutant can rescue the phenotype of sucrose sensitivity, indicating that *RtVP1* is a functional homolog of *AtVP1*.

### *RtVP1* overexpression enhances the growth and development of transgenic *Arabidopsis*

WT *Arabidopsis* overexpressing *RtVP1* exhibited apparent phenotypes compared to WT, *avp1* mutant, and *avp1* plants overexpressing *RtVP1* at different developmental stages. There was no significant difference was observed among WT, *avp1*, and transgenic seedlings at 3 days after sowing (**Figure 5A**). WT plants overexpressing *RtVP1* displayed faster shoot and root growth than WT and other plants after germination (**Figures 5B–F**). Subsequently, WT plants overexpressing *RtVP1* showed greater heights and growth rates than WT and other plants after transplanting to soil (**Figures 5G–J**). To assess the differences in phenotypes among these plants, the area, width, and length of the fifth leaf, internode distances, and plant height of 40-day-old plants, as well as the seedling fresh weight and root length of 14-day-old plants were measured (**Figure 6**). *RtVP1* overexpression in WT plants significantly increased the root length and fresh weight 2 weeks after sowing (**Figures 6A, B**). No significant differences were observed in the number of cauline branch, but significant differences in area, length, and width of fifth leaf, internode distance, height, and mature fresh weight were found between WT and WT plants overexpressing *RtVP1* after 40 days of growth (**Figures 6C–J**).

### *RtVP1* overexpression influences carbohydrate metabolism in transgenic *Arabidopsis*

**Figure 7:**
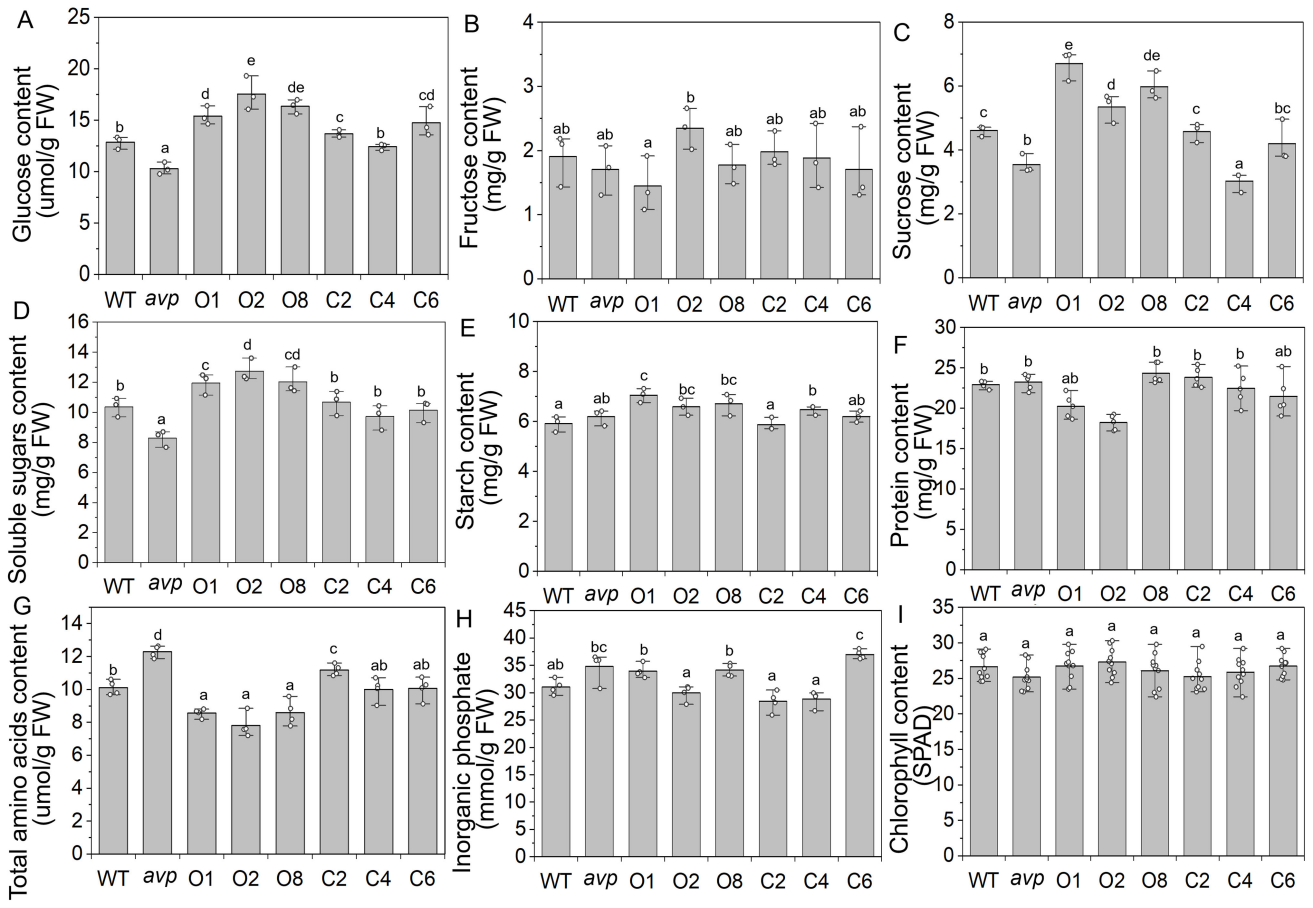
Physiological parameters detection of WT, *avp1*, and representative transgenic plants. **(A–E)** The glucose **(A)**, fructose **(B)**, sucrose **(C)**, soluble sugars **(D)**, and starch **(E)** were measured in WT, avp1, and representative transgenic plants grown 40 days after 4–5 h of illumination. **(F–I)** The protein **(F)**, total amino acid **(G)**, inorganic phosphate **(H)**, and chlorophyll content **(I)** were measured in rosettes of 40-day-old WT, *avp1*, and representative transgenic plants. Data are expressed as means ± SE. Values with the same letter are not significantly different [*P* < 0.05, Duncan’s multiple range test (MRT)].

A previous study has shown that *AtVP1* overexpression influences carbon partitioning in *Arabidopsis* plants (Khadilkar et al., 2016). To survey the effect of *RtVP1* overexpression on carbohydrate metabolism, the glucose, sucrose, fructose, soluble sugar, and starch content were measured in rosettes of WT, *avp1*, and representative transgenic plants grown for 40 days after 4 h of illumination (**Figure 7**). Compared with WT, transgenic *Arabidopsis* overexpressing *RtVP1* accumulated more soluble carbohydrates, such as glucose, sucrose, soluble sugars, and starch, but no significant differences in fructose were observed (**Figures 7A–E**). A recent study has shown that *AtVP1* overexpression enhances the nutrient and phosphate uptake of transgenic plants (Yang et al., 2015; Pizzio et al., 2015). To investigate the function of *RtVP1* in nutrient and phosphate uptake, the protein, amino acid, and inorganic phosphate content were measured in rosettes of transgenic plants and WT grown for 40 days. No consistent alterations in the protein content were observed between WT and transgenic plants, but significant decreases in the total amino acid content were observed (**Figures 7F, G**). There were no consistent changes in inorganic phosphate, although some lines had significantly increased in transgenic plants overexpressing *RtVP1* (**Figure 7H**). The chlorophyll content was measured in the fifth rosettes to assess photosynthetic capacity, and no significant differences were found between WT and transgenic plants (**Figure 7I**).

**Figure 8:**
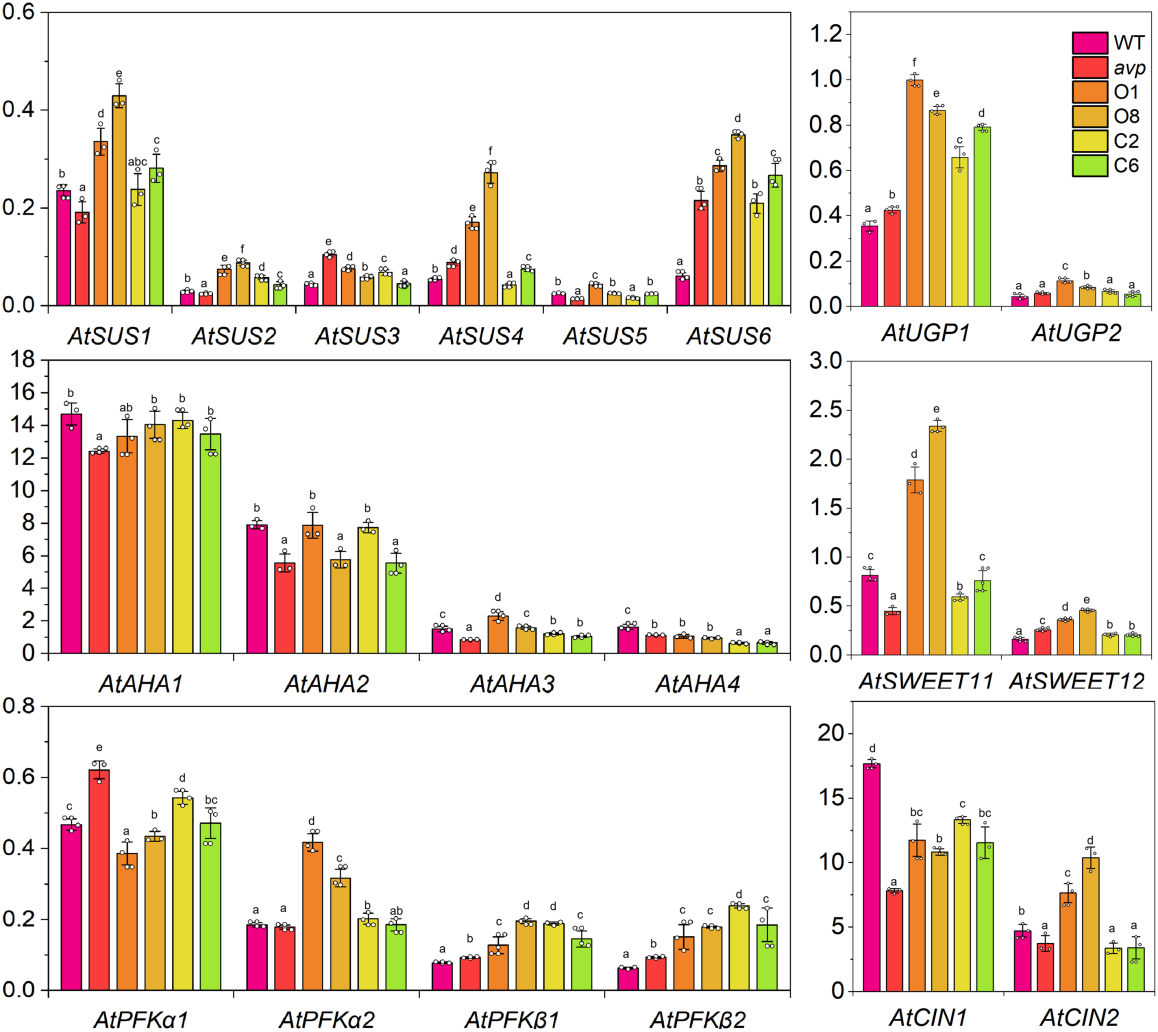
Detection of the expression levels of sucrose metabolism-related genes in WT, *avp1*, and representative transgenic plants (O1, O8, C2, and C6). WT, *avp1*, and representative transgenic plants were grown in transplanting boxes for 40 days after 4–5 h of illumination. Data are expressed as means ± SE. Values with the same letter are not significantly different [*P* < 0.05, Duncan’s multiple range test (MRT)].

To survey the effect of *RtVP1* overexpression on sucrose metabolism, real-time PCR was performed to assess the transcript abundance of some sucrose metabolism-related genes (**Figure 8**). The results showed that sucrose synthase genes (*AtSUS1*, *AtSUS2*, *AtSUS4*, and *AtSUS6*), UDP-Glc pyrophosphatases (*AtUGP1* and *AtUGP2*), phosphofructokinases (*AtPFKα2*, *AtPFKβ1*, and *AtPFK β2*), SWEETs involved in phloem loading (*AtSWEET11* and *AtSWEET12*), and cytosolic alkaline invertases (*AtCIN2*) were upregulated in transgenic *Arabidopsis* overexpressing *RtVP1* (**Figure 8**). However, plasma membrane H^+^-ATPases (*AtAHA1*, *AtAHA2*, *AtAHA3*, and *AtAHA4*) showed no consistent changes in transgenic *Arabidopsis* (**Figure 8**).

### *RtVP1* overexpression enhances salt tolerance in transgenic *Arabidopsis*

**Figure 9:**
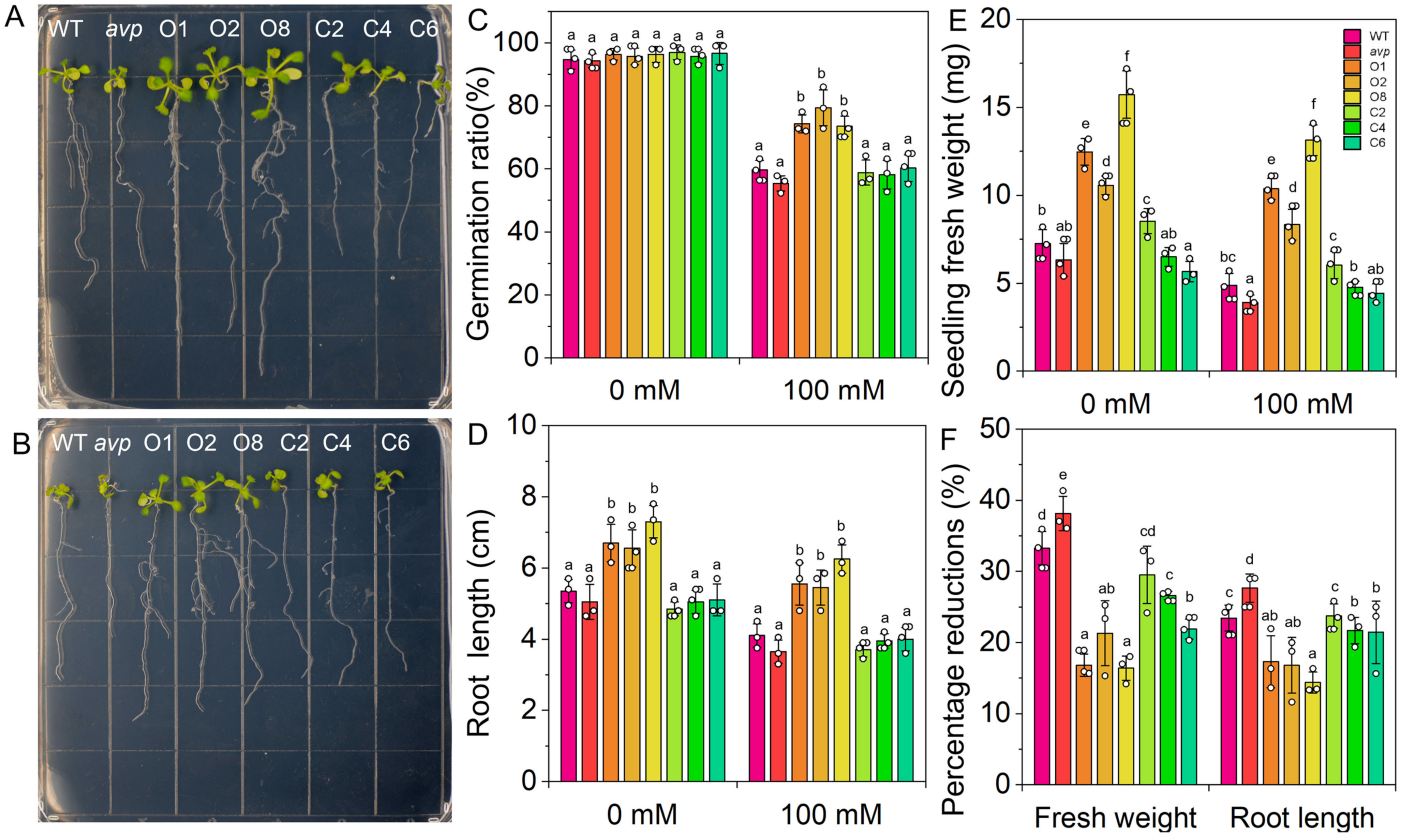
Seeds of WT, *avp1*, and transgenic plants were germinated on MS medium and transferred to MS containing 0 **(A)** and 100 mM NaCl **(B)** to investigate differences in root length. The seed germination of WT, *avp1*, and transgenic plants were grown on MS medium with 0 and 100 NaCl treatments for 7 days after sowing **(C)**. Comparative measurements of primary root length **(D)** and seedling fresh weight **(E)**. The percentage reduction of root length and seedling fresh weight (%) compared with the unstressed control **(F)**. Data are expressed as means ± SE. Values at each NaCl concentration with the same letter are not significantly different [*P* < 0.05, Duncan’s multiple range test (MRT)].

**Figure 10:**
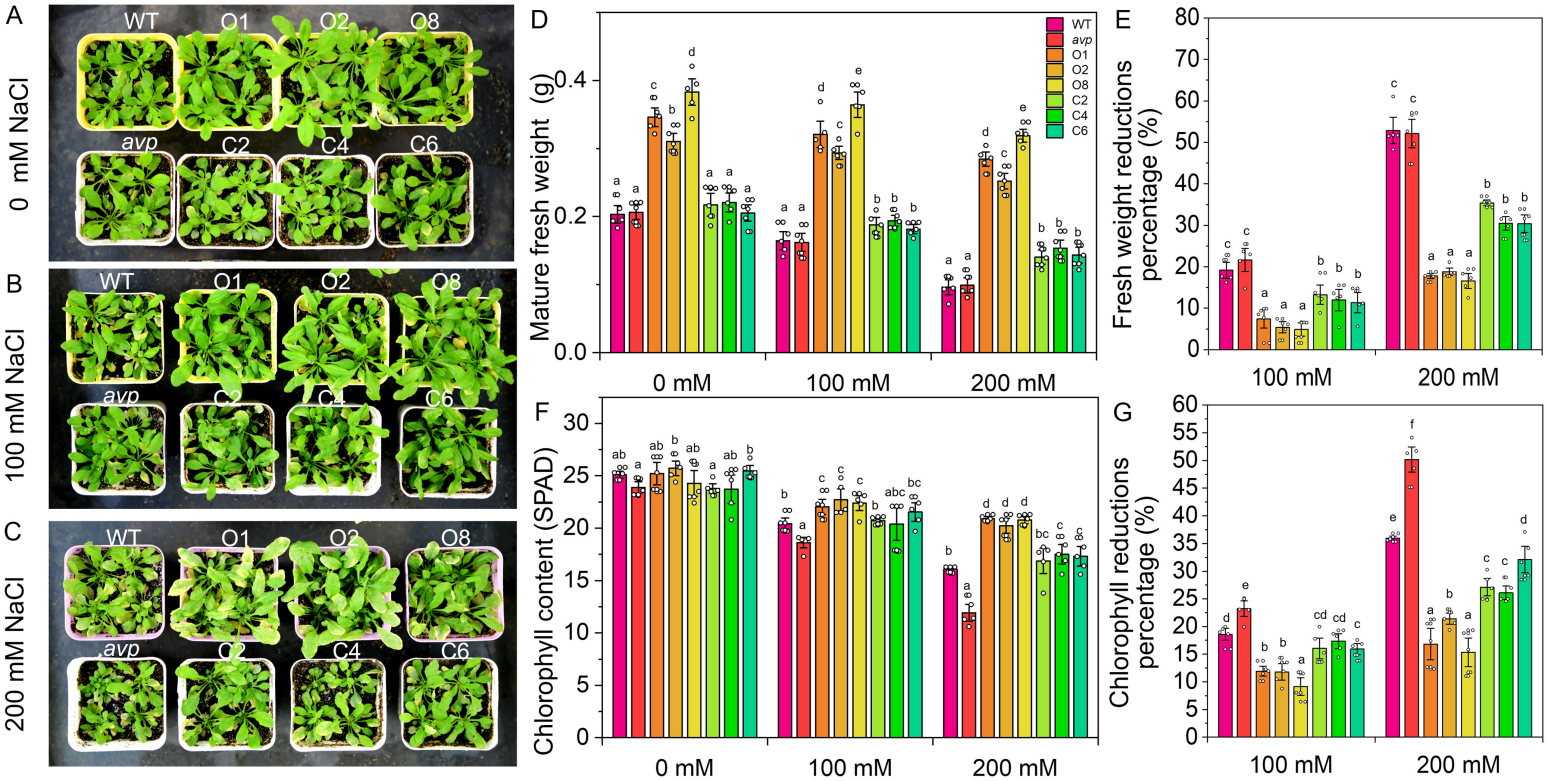
Effects of *RtVP1* expression on transgenic *Arabidopsis thaliana* in response to salt stress. Thirty-day-old WT, *avp1*, and transgenic plants grown on soil were treated with distilled water [0 mM NaCl **(A)**] and various concentrations of NaCl [100 mM and 200 mM **(B, C)**] for 10 days. The shoot fresh weight **(D)** and chlorophyll content **(F)** of leaves from WT and transgenic *Arabidopsis* were measured in response to treatment with 100 mM and 200 mM NaCl. The percentage reduction of shoot fresh weight **(E)** and chlorophyll content **(G)** compared with the non-stressed control. Data are expressed as means ± SE. Values at each NaCl concentration with the same letter are not significantly different [*P* < 0.05, Duncan’s multiple range test (MRT)].

To assess the salt tolerance of *RtVP1*-overexpressing plants, the germination rate, root length, and fresh weight of seedlings were measured under salt stress conditions (**Figure 9**). The germination rate of *RtVP1*-overexpressing seeds was obviously higher than that of WT under salt stress conditions, but no apparent alterations were observed under normal conditions (**Figure 9C**). Transgenic seedlings overexpressing *RtVP1* exhibited a higher root length and fresh weight than WT under normal or salt stress conditions (**Figures 9D, E**), but the percentage reduction relative to normal conditions was lower than that of WT under 100 mM NaCl treatment (**Figure 9F**). To further evaluate the salinity tolerance conferred by *RtVP1*, 30-day-old transgenic *A*. *thaliana* was treated with 0 mM, 100 mM, and 200 mM NaCl for 10 days (**Figures 10A–C**). The mature fresh weight of *RtVP1*-overexpressing *Arabidopsis* was significantly higher than that of WT under normal and salt stress conditions (**Figure 10D**), but the percentage reduction relative to normal conditions was lower than that of WT under salt stress (**Figure 10E**). The chlorophyll content was significantly increased in transgenic plants under salt stress compare with that of WT, and the percentage reduction was lower than that of WT (**Figures 10F, G**).

**Figure 11:**
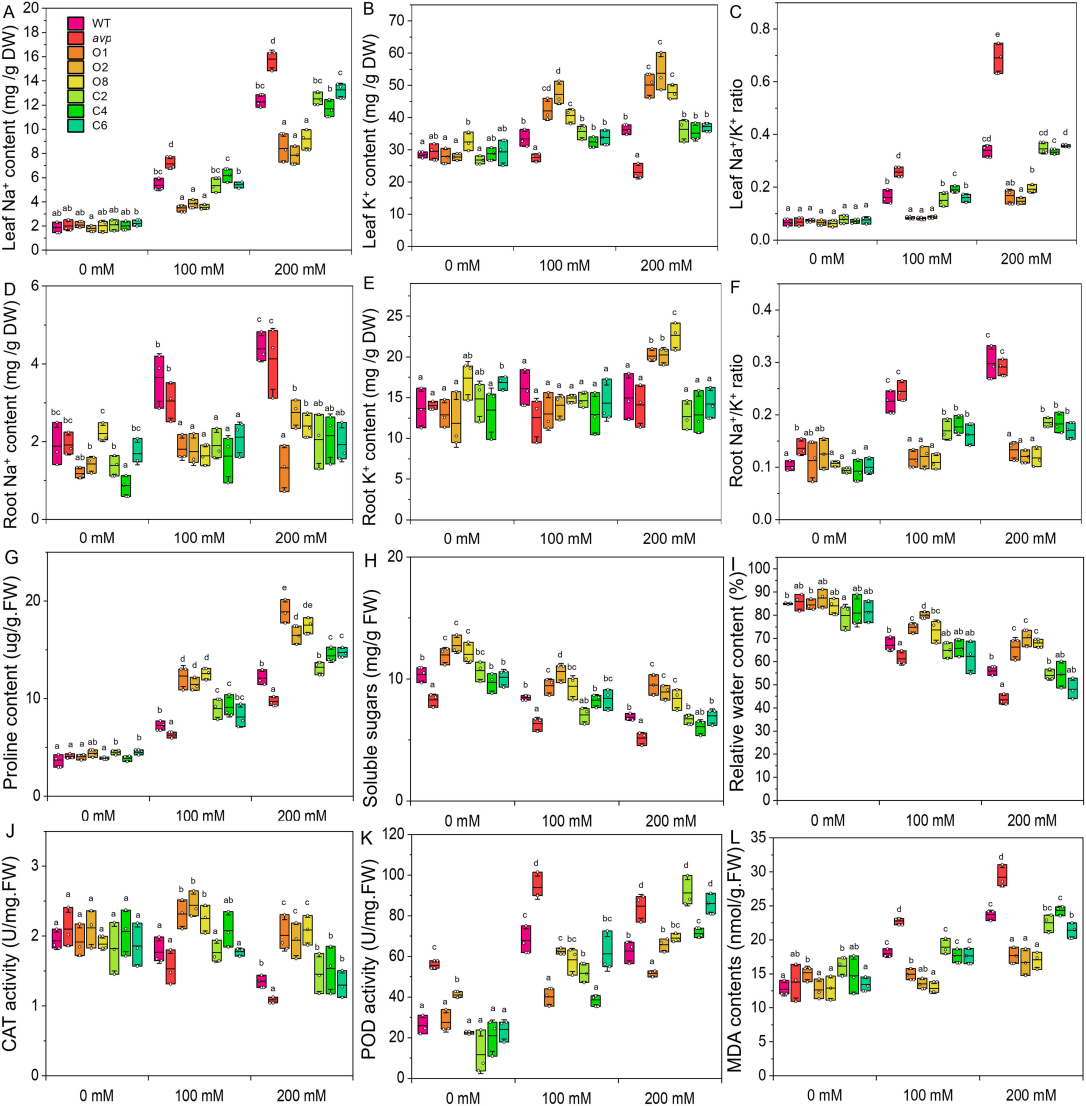
The Na^+^ **(A, D)** and K^+^ **(B, E)** content of WT, *avp1*, and transgenic *Arabidopsis* were measured in rosette leaves **(A, C)** and roots **(B, D)** under various concentrations of NaCl (0 mM, 100 mM, and 200 mM NaCl), and Na^+^/K^+^ ratios were calculated for leaves **(C)** and roots **(F)**. Proline **(G)**, soluble sugar **(H)**, and relative water content **(I)** were detected in the rosette leaves of all lines in response to treatment with 100 mM and 200 mM NaCl. The activities of catalase [CAT **(J)**] and peroxidase [POD **(K)**], as well as malondialdehyde [MDA **(L)**], were measured in the rosette leaves of all lines in response to treatment with 100 mM and 200 mM NaCl. Data are expressed as means ± SE. Values at each NaCl concentration with the same letter are not significantly different [*P* < 0.05, Duncan’s multiple range test (MRT)].

### Overexpression of *RtVP1* in *Arabidopsis* improves the capacity of Na^+^/K^+^ homeostasis regulation, osmoregulation, and production of antioxidant responses under salt stress

To investigate the role of *RtVP1*, the Na^+^ and K^+^ contents of the leaves and roots were measured in WT, *avp1*, and representative transgenic plants. In leaves, *RtVP1* overexpression in WT plants exhibited higher K^+^ and lower Na^+^ and Na^+^/K^+^ ratio than WT under salt stress, and transgenic *avp1* mutants also showed the higher K^+^ and lower Na^+^ and Na^+^/K^+^ ratio than *avp1* mutants; but no significant differences were found under normal condition (**Figures 11A–C**). In roots, transgenic plants had a lower Na^+^ and Na^+^/K^+^ ratio than WT or *avp1* mutants, and no consistent pattern in the K^+^ content was found under salt stress (**Figures 11D–F**). These results indicate that *RtVP1* maintains the leaf and root homeostatic Na^+^/K^+^ balance under salt stress.

With increasing NaCl concentrations, the proline content was elevated sharply in all lines, but the soluble sugar content and RWC declined slowly (**Figures 11G–I**). WT plants overexpressing *RtVP1* accumulated more proline and maintained a higher RWC than WT, *avp1*, and *avp1* mutant plants overexpressing *RtVP1* under NaCl treatment, but no significant differences were observed among them under normal conditions (**Figures 11G–I**). Interestingly, the soluble sugar content of WT plants overexpressing *RtVP1* was significantly higher than that of any other lines under normal or salt stress conditions (**Figure 11H**), but the percentage reduction relative to normal conditions was lower than that of other lines (data not shown). WT plants overexpressing *RtVP1* had a higher CAT activity and lower MDA content than WT, *avp1*, and transgenic *avp1* plants under salt stress (**Figures 11J, L**); and no differences was found in POD activity (**Figure 11K**). These results indicate that *RtVP1* can improve the osmotic and antioxidant regulatory abilities of transgenic plants in response to salt stress.

### *RtVP1* enhances the expression of stress-related genes in transgenic *Arabidopsis* lines under salt stress conditions

**Figure 12:**
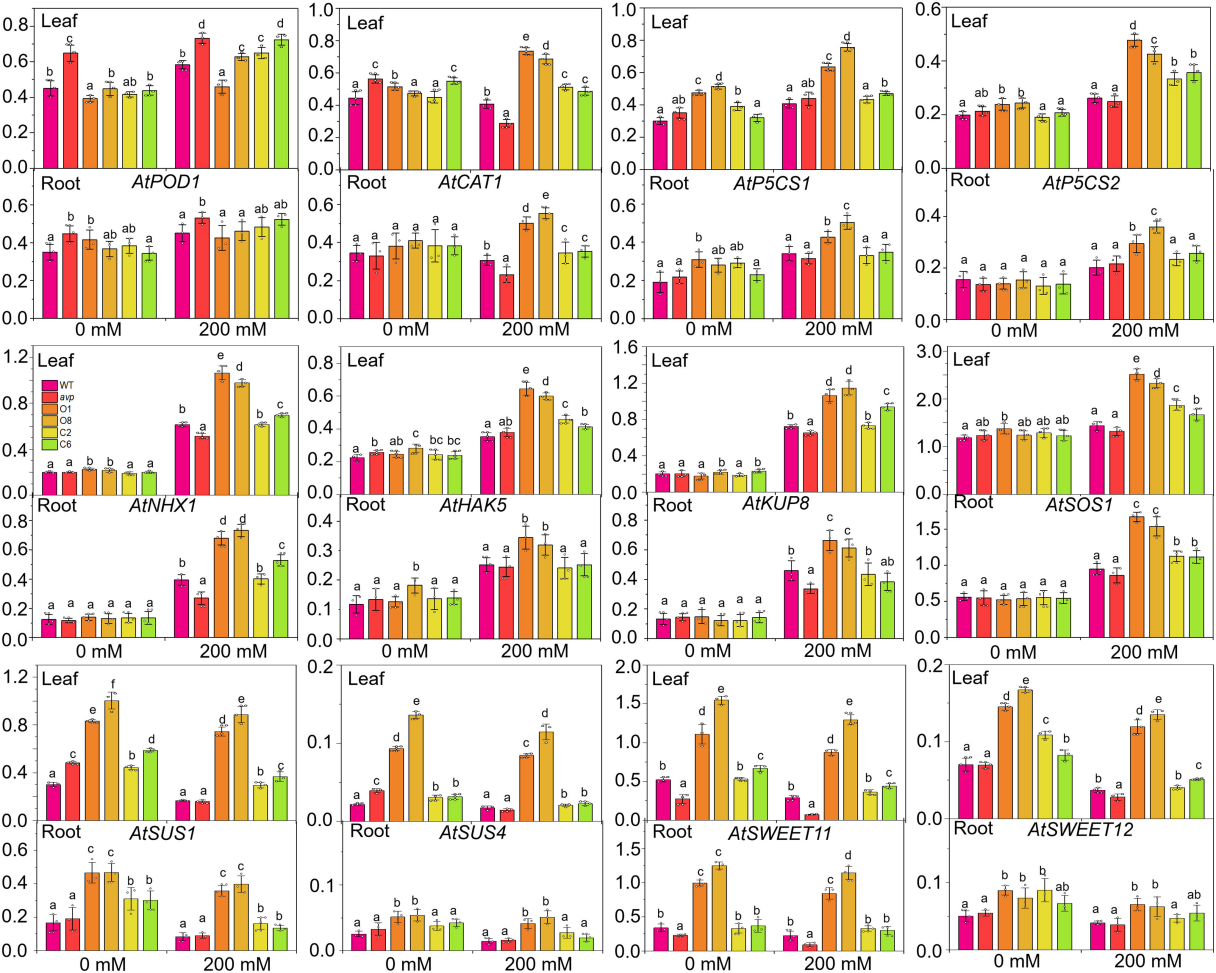
Relative expression levels of stress-response genes in WT, *avp1*, and transgenic *Arabidopsis*. WT, *avp1*, and transgenic *Arabidopsis* were grown for 30 days in soil boxes and treated with distilled water (0 mM NaCl) or 200 mM NaCl for 10 d. Data are expressed as means ± SE. Values at each NaCl concentration with the same letter are not significantly different [*P* < 0.05, Duncan’s multiple range test (MRT)].

To further analyze the salinity tolerance conferred by *RtVP1*, the expression of genes relate to salt stress were investigated in leaves and roots of WT, *avp1*, and transgenic *Arabidopsis*. The ion transport-related genes, such as plasma membrane salt-overly sensitive 1 (*AtSOS1*), vacuolar Na^+^/H^+^ antiporter (*AtNHX1*), K^+^ uptake transporters 8 (*AtKUP8*), and high-affinity K^+^ transporter 5 (*AtHAK5*), were also markedly stimulated in leaves and roots (**Figure 12**), indicating that *RtVP1* maintained the Na^+^/K^+^ homeostatic balance in transgenic *Arabidopsis* by ion transporter synergism in response to salt stress. Furthermore, proline biosynthesis genes (*AtP5CS1* and *AtP5CS2*) and sucrose metabolism-related genes (*AtSUS1*, *AtSUS3*, *AtSUS4*, *AtSWEET11*, and *AtSWEET12*) were significantly upregulated in leaves and roots of WT *Arabidopsis* overexpressing *RtVP1* compared to that of WT under salt stress (**Figure 12**), implying that *RtVP1* overexpression improved osmoregulation in transgenic *Arabidopsis* through reinforced expression of proline biosynthesis genes and sucrose metabolism-related genes in response to salt stress, thus enhancing the salt tolerance to transgenic *Arabidopsis*. Additionally, a CAT gene (*AtCAT1*) was also upregulated in leaves and roots, indicating that *RtVP1* improved antioxidant regulation in transgenic *Arabidopsis*.

### Functional analysis of the *RtVP1* gene in yeast

**Figure 13:**
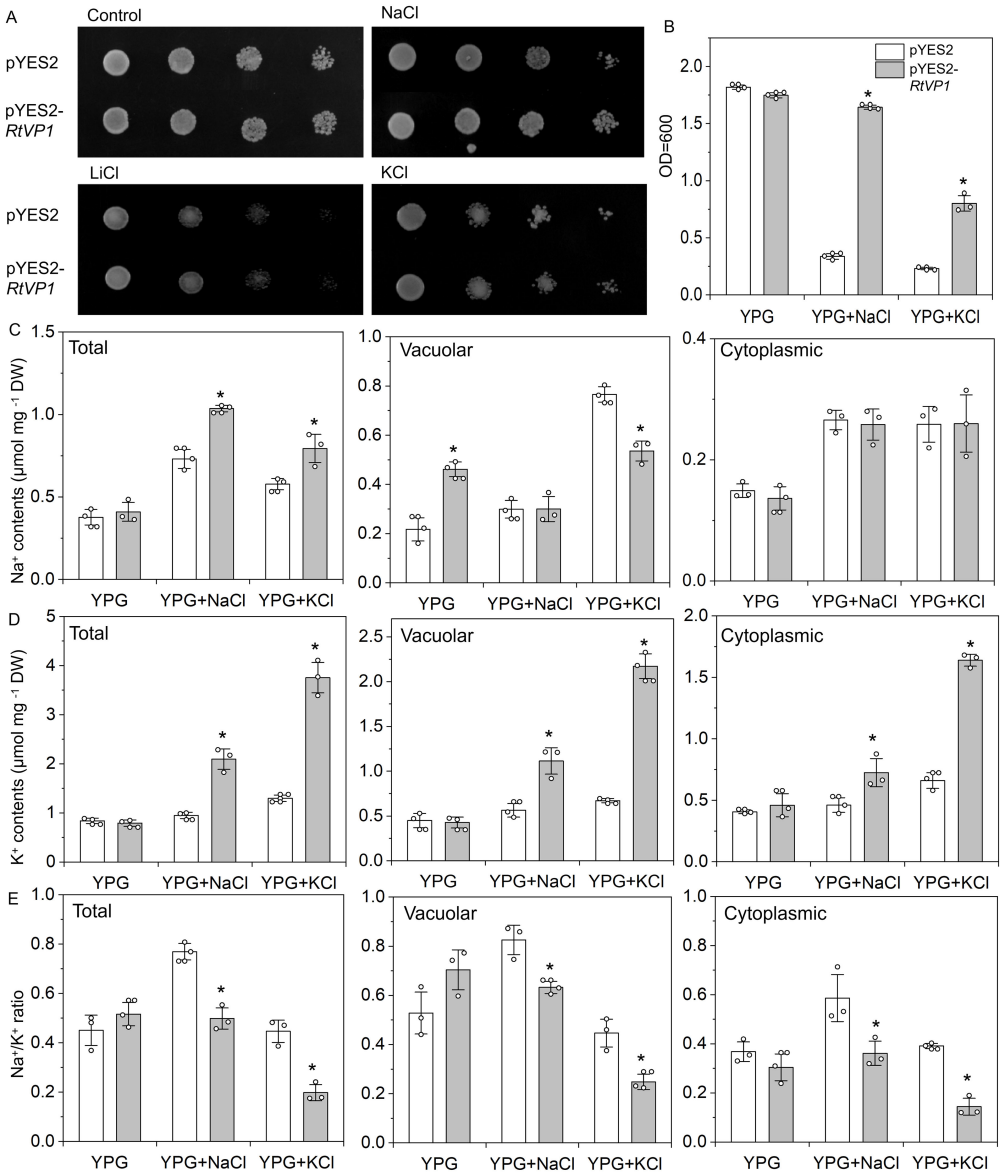
Expression of *RtVP1* in the *AXT3* yeast mutant strain. Yeast cells harboring the pYES2 empty vector were used as controls, and a yeast strain expressing pYES2-*RtVP1* was used to assess salt tolerance. **(A)** A total of 5 µL from each 10-fold serial dilution was spotted onto yeast extract-peptone-glycerol (YPG) medium supplemented as follows: Control (no additions), NaCl (100 mM), KCl (1 M), and LiCl (20 mM). **(B)** The cells were grown in liquid YPG with or without 100 mM NaCl or 1 M KCl, and OD_600 nm_ was measured after shaking for 48 h at 30°C. The total intracellular Na^+^ **(C)** and K^+^ **(D)** content as well as the Na^+^/K^+^ ratio **(E)** were determined in cells grown on 100 mM NaCl or 1 M KCl. Values are expressed as means ± SE. An asterisk (*) indicates a significant difference (*P* < 0.05, Duncan’s multiple range test: pYES2 versus pYES2-*RtVP1*).

To investigate the function of *RtVP1* in yeast, pYES2-RtVP1 vectors were introduced into the salt-sensitive yeast mutant *AXT3* strain (△ena1::HIS3::ena4 △nha1::LEU2 △nhx1::KanMX ura3-1 can1-100 leu2-3112 trp1-1 his3-11), and the Na^+^ and K^+^ contents were determined under salt stresses. No significant differences were observed between transgenic yeast expressing *RtVP1* and control under normal conditions. However, *RtVP1*-transgenic lines showed a better growth than control under 100 mM NaCl, 1 M KCl, or 20 mM LiCl (**Figures 13A, B**). Under normal conditions, the Na^+^ and K^+^ contents and Na^+^/K^+^ ratio in intracellular, vacuolar, and cytoplasmic were not apparently different between transgenic yeast transformed with pYES2-RtVP1 and pYES2, except for Na^+^ accumulation in vacuoles, which increased. Under salt stress conditions (100 mM NaCl or 1 M KCl), transgenic lines expressing *RtVP1* had higher Na^+^ and K^+^ contents and lower Na^+^/K^+^ ratio in intracellular that control. Higher K^+^ accumulation and unchanged Na^+^ led to the lower Na^+^/K^+^ ratio in cytoplasmic of transgenic lines expressing *RtVP1*. In vacuolar, Na^+^ content of transgenic yeast did not change under 100 mM NaCl but significantly decreased under 1 M KCl, and transgenic yeast had a higher K^+^ and a lower Na^+^/K^+^ ratio than control under salt stresses (**Figure 13C**). These results suggest that RtVP1 can enhance salt tolerance in transgenic yeast by maintaining a relatively lower Na^+^/K^+^ ratio in the cell in response to salt stress.

## Discussion

### Enhanced vegetative growth of transgenic *Arabidopsis* may be attributed to the involvement of RtVP1 in sucrose metabolism and carbohydrate accumulation

Vacuolar H^+^-PPases can enhance plant vegetative growth and enhance plant shoot and root biomass accumulation. *AtVP1* overexpression increased the rosette leaf area and number and root dry weight of transgenic *Arabidopsis*, and the number of epidermal cells contained in fixed areas did not change between WT and *avp1 Arabidopsis*, suggesting that the larger organs have an increased cell number (Li et al., 2005). Transgenic *Agrostis stolonifera* expressing *AtVP1* exhibited better shoot growth and more robust root development and maintained a higher chlorophyll a and b content than WT plants, indicating the role of improved photosynthesis in enhanced growth performance (Li et al., 2010). *TaVP1* overexpression enhanced the root length, shoot and root dry weight, and height and elevated the photosynthetic rate in transgenic tobacco, although the chlorophyll content showed no change (Gouiaa et al., 2012; Li et al., 2014). Overexpression of *MtVP1* from *Medicago truncatula* improved the development of the root system, accelerated the formation and growth of young leaflets, and delayed the flowering time by approximately 2 weeks in transgenic *Arabidopsis* and potato, indicating that *MtVP1* can promote the vegetative growth of plants and result in a higher crop yield (Wang et al., 2014). In the present work, *RtVP1* overexpression increased the area, length, and width of the fifth rosette leaf, internode distance, plant height, root length, and seedling fresh weight of 14-day-old plants and enhanced the vegetative growth in transgenic plants at different developmental stages. However, the chlorophyll content did not change between WT and transgenic plants, which agrees with results from transgenic tobacco expressing *TaVP1* from *Triticum aestivum* (Gouiaa et al., 2012). This implied that the enhancement of vegetative growth of transgenic Arabidopsis expressing *RtVP1* may not be caused by the increased chlorophyll content promoting photosynthesis.

Recent reports have shown that plant vacuolar H^+^-PPases participate in the metabolism of polysaccharides to enhance the vegetative growth of plants. For example, *MtVP1* overexpression increased the sucrose concentration in buds and tubers of transgenic potato and enhanced the vegetative growth of shoots and roots (Wang et al., 2014). Transgenic *Beta vulgaris* co-expressing *ZxNHX1* and *ZxVP1-1* exhibited a higher sucrose, fructose, and glucose content in storage roots than WT plants and had a higher fresh and dry weight of leaves and petioles under normal conditions (Wu et al., 2015). Similarly, in the present work, *RtVP1* overexpression increased the glucose, sucrose, and soluble sugar content and improved the vegetative growth in transgenic plants at different developmental stages, indicating that *RtVP1* participated in sucrose metabolism to improve vegetative growth (**Figures 5**–**7**). These results support the model proposed by Khadilkar et al. (2016). AtVP1 on the vacuolar membrane of mesophyll cells enhances the hydrolysis of PPi, which is the product of sucrose synthesis; thus, hydrolysis of PPi accelerates sucrose synthesis. Furthermore, AtVP1 on the plasma membrane of companion cells uses proton motive force to synthesize PPi, which makes sucrose oxidation accelerate and production of more ATP, and then produce more proton motive force by P-type ATPases. Thus, *AtVP1* plays a key role in maintaining PPi homeostasis and enhancing phloem loading and long-distance transport of photosynthates (Pizzio et al., 2015; Khadilkar et al., 2016). Some lines of transgenic *Arabidopsis* expressing *RtVP1* accumulated more Pi compared with WT plants under normal conditions, which is consistent with results from transgenic *Arabidopsis* (Khadilkar et al., 2016) and *Agrostis stolonifera* (Li et al., 2010), indicating that *RtVP1* may be involved in hydrolysis of PPi and maintain PPi homeostasis in transgenic plants. In addition, some sucrose metabolism-related genes, such as sucrose synthase genes (*AtSUS1*, *AtSUS2*, *AtSUS4*, and *AtSUS6*), UDP-Glc pyrophosphatases (*AtUGP1* and *AtUGP2*), phosphofructokinases (*AtPFKa2*, *AtPFK b1*, and *AtPFK b2*), and SWEETs involved in phloem loading (*AtSWEET11* and *AtSWEET12*), were upregulated in transgenic *Arabidopsis* expressing *RtVP1*. These results further indicate that *RtVP1* might be involved in various physiological processes, such as sucrose synthesis, sucrose oxidation, sucrose phloem loading, and long-distance transport, thereby improving the vegetative growth of transgenic plants and accelerating biomass accumulation.

### Salt tolerance of transgenic plants may be due to RtVP1 effectively maintaining plant ion homeostasis and improving the capacity of antioxidant regulation and osmotic adjustment

Plant vacuolar H^+^-PPases promote proton pumping across the membrane into the vacuole to create a proton motive force, which provides a motive source for energizing NHXs to maintain the Na^+^-K^+^ homeostatic balance in response to salt stress. For example, *PtVP1*.*1* overexpression resulted in more K^+^ and less Na^+^ accumulation in leaves of transgenic poplar, an increase in root Na^+^ efflux and H^+^ influx, and upregulation of the *PtNHX2* and *PtNHX4* genes, indicating that *PtVP1*.*1* improved the Na^+^/H^+^ exchange activity in transgenic plants as a result of both enhanced expression and activity of Na^+^/H^+^ transporters, thus maintaining the lower Na^+^/K^+^ ratio to confer salt tolerance of plants (Yang et al., 2015). Our previous work demonstrated that *RtNHX1* conferred yeast salt tolerance and sequesters more Na^+^ and K^+^ in the vacuole to maintain a lower Na^+^/K^+^ ratio in transgenic yeast cells (Li et al., 2017). However, the Na^+^ and K^+^ distribution patterns of transgenic yeast cells expressing *RtVP1* were similar to those of *RtNHX1*, implying that increased Na^+^ and K^+^ in *RtVP1* transgenic yeast cells may be due to the promotion of NHX activity by *RtVP1* expression, which causes Na^+^ and K^+^ to be sequestered in the vacuole. In addition, transgenic *Arabidopsis* expressing *RtVP1* accumulated more K^+^ and less Na^+^ in leaves related to WT plants under salt stress, as well as in transgenic tobacco expressing *TaVP* (Gouiaa et al., 2012) and transgenic poplar expressing *PtVP1*.*1* (Yang et al., 2015). The less Na^+^ content in the leaves of transgenic plants may be due to plasma membrane Na^+^/H^+^ antiporters (e.g., *AtSOS1*) excreting Na^+^ from the cytoplasm to prevent the Na^+^ loading into the roots and remove excess Na^+^ from the leaves and vacuolar Na^+^/H^+^ transporters (e.g., *AtNHX1*) sequestering Na^+^ into the vacuole in roots to reduce long-distance Na^+^ transport from roots to shoots (**Figure 12**). The more K^+^ accumulation in the leaves of transgenic plants may be attributed to the significantly upregulated expression of high-affinity K^+^ transporter genes (e.g., *AtHAK5*) and K^+^ uptake transporter (e.g., *AtKUP8*) (**Figure 12**). These results indicated that *RtVP1* can maintain and reconstruct the Na^+^-K^+^ homeostasis balance through multiple ion transporters synergetic regulation, thereby improving the salt tolerance of transgenic plants.

Previous studies have demonstrated that vacuolar H^+^-PPases can enhance the capacity for osmotic adjustment and antioxidant regulation to confer plant salt tolerance. For example, efficient osmotic adjustment of transgenic plants overexpressing H^+^-PPase genes is reflected in better RWC and proline and soluble sugar accumulation (Li et al., 2010; Wu et al., 2015; Meng et al., 2017; Yu et al., 2017), whereas efficient ROS scavenging was mainly manifested by increased antioxidant enzyme activity and expression and decreased MDA content (Gouiaa et al., 2012; Anjaneyulu et al., 2014; Li et al., 2014; Yu et al., 2017). Xue et al. (2012a, b) reported that the content of proline, soluble sugar, and free amino acid and the activities of SOD and POD were significantly increased in *R. trigyna* under salt stress, and the MDA and relative electrolytic leakage were significantly decreased. Dang et al. (2013) demonstrated that the expression of genes related to ROS scavenging (298) and osmotic adjustment (129) were significantly increased in *R*. *trigyna* under salt stress. Our previous work showed that transgenic plants overexpressing *RtNHX1* and *RtHKT1* exhibited higher salt tolerance than WT plants and had a higher antioxidant enzyme activity, RWC, and proline content (Li et al., 2017, 2019). In the present work, *RtVP1* overexpression increased root development, chlorophyll content, and fresh plant weight under salt stress (**Figures 9**, **10**). This is consistent with previous reports by Gouiaa et al. (2012) and Anjaneyulu et al. (2014). Moreover, the increased proline and soluble sugar content, RWC, and CAT activity and the decreased MDA content were observed in transgenic plants under salt stress. The expression of sucrose metabolism-related genes (*AtSUS1*, *AtSUS3*, *AtSUS4*, *AtSWEET11*, and *AtSWEET12*), proline synthesis genes (*AtP5CS1* and *AtP5CS2*), and an antioxidant enzyme gene (*AtCAT1*) was also stimulated (**Figure 12**). Therefore, the *R*. *trigyna* vacuolar H^+^-PPases can improve the osmoregulation and antioxidant regulation ability, and then endow transgenic plant with salt tolerance.

Thus far, many vacuolar H^+^-PPase genes from different species have been cloned and transformed into plants for functional investigation. Most of the current research has demonstrated that overexpression of vacuolar H^+^-PPase genes can improve plant salt tolerance through the analyses of growth status and Na^+^ and K^+^ distribution under salt stress conditions (Gaxiola et al., 2001; Gao et al., 2006; Brini et al., 2007; Lv et al., 2008; Li et al., 2010; Meng et al., 2017). However, the molecular regulatory mechanisms underlying the increase in plant salt tolerance have not been fully elucidated in these studies. Transgenic plants overexpressing H^+^-PPase genes from different species had different Na^+^/K^+^ distribution in leaves. For example, under salt stress conditions, *AtVP1* overexpression resulted in a higher Na^+^ and K^+^ accumulation in transgenic *Arabidopsis* (Gaxiola et al., 2001), creeping bentgrass (Li et al., 2010), transgenic *Arabidopsis* overexpressing *ZmVP1* (Chen et al., 2015) and *AnVP1* (Yu et al., 2017), transgenic finger millet overexpressing *SbVPPase* (Anjaneyulu et al., 2014), transgenic sugar beet overexpressing *ZxVP1-1* (Wu et al., 2015), and transgenic tobacco overexpressing *IlVP* (Meng et al., 2017) compared with that of control plants. However, our work showed that the decreased Na^+^ and increased K^+^ in *RtVP1* overexpressing transgenic *Arabidopsis* leaves were found under salt stress conditions (**Figure 11**), which is consistent with transgenic poplar overexpressing *PtVP1*.*1* (Yang et al., 2015) and transgenic tobacco overexpressing *TaVP* (Gouiaa et al., 2012). This characteristic of Na^+^-K^+^ distribution may cause changes in multiple metabolic pathways in plants. For example, the increased CAT activity, proline content, and RWC and the decreased MDA were observed in transgenic plant under salt stress. The expression of *AtCAT1*, *AtP5CS1*, and *AtP5CS2* was also increased in roots and leaves of transgenic *Arabidopsis* (**Figure 12**). Meanwhile, RtVP1 is involved in sucrose metabolism and accumulates more soluble sugars in response to salt stress. This resulted in a higher salt tolerance in transgenic plants compared to WT plants. These results implied that overexpression of *RtVP1* promoted the accumulation of soluble sugars in transgenic plants, reconstructed the Na^+^-K^+^ homeostasis balance, and then improved the osmoregulation and antioxidant regulation ability, thus improving the salt tolerance of transgenic plants.

## Conclusion

In present study, the H^+^-pyrophosphatase gene was isolated from *R. trigyna* (*RtVP1*) that encoded a plasma membrane and tonoplast-localized protein. *RtVP1* was functionally homologous to *AtVP1* and can rescue the sucrose-sensitive deficiencies of the *avp1* mutant. Under normal conditions, *RtVP1* can regulate sucrose metabolism and carbohydrate accumulation, thereby significantly promoting the vegetative growth of transgenic *Arabidopsis*. Under salt stress conditions, *RtVP1* can promote ion segregation into vacuole, maintain a low Na^+^/K^+^ ratio, and reconstruct Na^+^ and K^+^ homeostasis, which can also promote the accumulation of osmoregulatory substances and the activity of reactive oxygen species scavenging systems, thereby conferring salt tolerance in transgenic plants.

## Data Availability

The original contributions presented in the study are publicly available. This data can be found here: NCBI, PP816023.

## Supplementary material

Supplementary Figure 2 The phylogenetic tree of plant H^+^-PPase sequences, constructed as described in the methods section. The following protein sequences are included: *Populus trichocarpa* (PtVP1.1, XP_002331062.1; PtVP1.2, XP_002325187.1; PtVP1.3, XP_002318956.1; PtVP1.4, XP_002330249.1), *Arabidopsis thaliana* (AtVP1, NP_173021.1; AtVP2, AAF31163.1), *Brassica napus* (BnVP1, AGJ81362.1; BnVP2, XP_013650109.1), *Camelina sativa* (CsVP1, XP_010476656.1; CsVP2, XP_010417506.1), *Prunus persica* (PpVP1, AAL11506.1), *Salicornia europaea* (SeVP1, AEI17666.1), *Nicotiana tabacum* (NtVP1, NP_001312147.1), *Gossypium hirsutum* (GhVP1, NP_001313687.1), *Glycine max* (GmVP1, XP_003528302.1), *Prunus mume* (PmVP1, XP_008228676.1), *Beta vulgaris* subsp. *vulgaris* (BvVP1, XP_010691127.1), and *Sesamum indicum* (SiVP1, XP_011073387.1).
