# Supplementary figures and images for: Overexpression of vacuolar H+-pyrophosphatase from a recretohalophyte Reaumuria trigyna enhances vegetative growth and salt tolerance in transgenic Arabidopsis thaliana
Source: Front Plant Sci. 2024 Nov 13;15:1435799. doi: 10.3389/fpls.2024.1435799 (PMC11598511; doi:10.3389/fpls.2024.1435799)

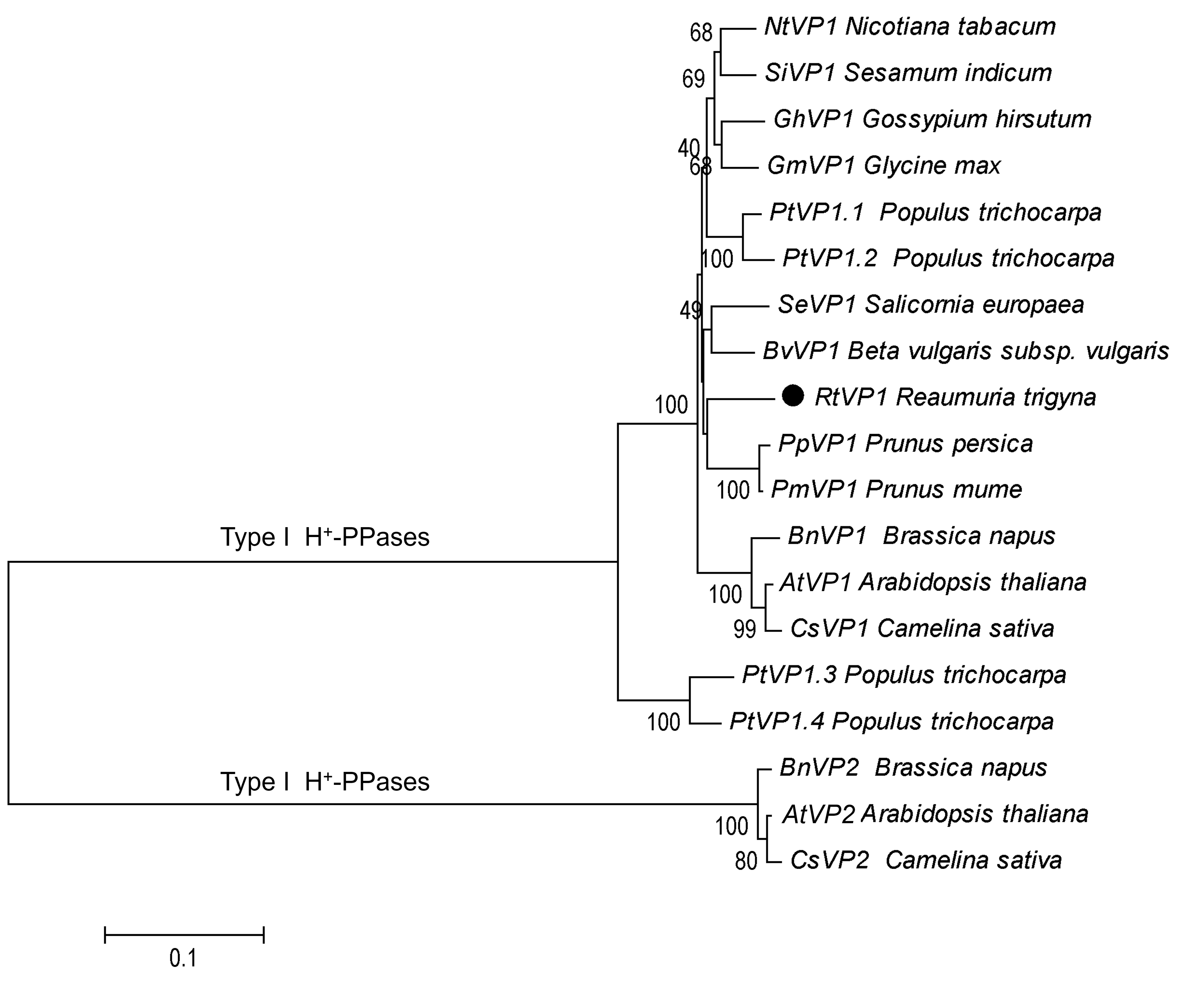

Supplement: Supplementary file 1 [file Image1.tif]

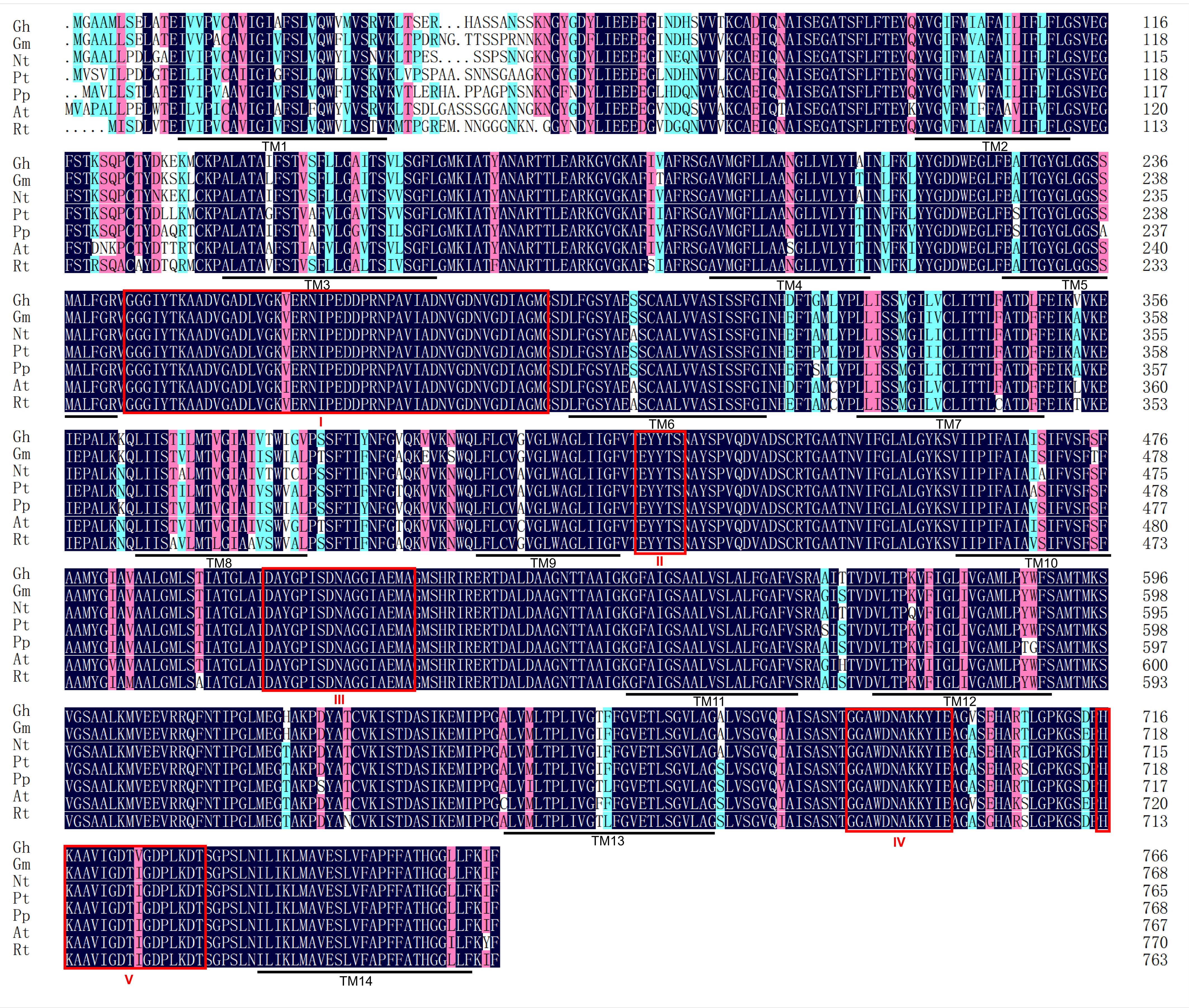

Supplement: Supplementary Figure 2 — The phylogenetic tree of plant H+-PPase sequences, constructed as described in the methods section. The following protein sequences are included: Populus trichocarpa (PtVP1.1, XP_002331062.1; PtVP1.2, XP_002325187.1; PtVP1.3, XP_002318956.1; PtVP1.4, XP_002330249.1), Arabidopsis thaliana (AtVP1, NP_173021.1; AtVP2, AAF31163.1), Brassica napus (BnVP1, AGJ81362.1; BnVP2, XP_013650109.1), Camelina sativa (CsVP1, XP_010476656.1; CsVP2, XP_010417506.1), Prunus persica (PpVP1, AAL11506.1), Salicornia europaea (SeVP1, AEI17666.1), Nicotiana tabacum (NtVP1, NP_001312147.1), Gossypium hirsutum (GhVP1, NP_001313687.1), Glycine max (GmVP1, XP_003528302.1), Prunus mume (PmVP1, XP_008228676.1), Beta vulgaris subsp. vulgaris (BvVP1, XP_010691127.1), and Sesamum indicum (SiVP1, XP_011073387.1). [file Image2.tif]
