# Supplementary material for: Overexpression of vacuolar H+-pyrophosphatase from a recretohalophyte Reaumuria trigyna enhances vegetative growth and salt tolerance in transgenic Arabidopsis thaliana
Source: Front Plant Sci. 2024 Nov 13;15:1435799. doi: 10.3389/fpls.2024.1435799 (PMC11598511; doi:10.3389/fpls.2024.1435799)
Supplement: Supplementary file 3 [file Table1.doc]

Table S1

List of the primers used for the experiments.

| 5'-ACTGTTGAAATCGCTGCTCGGCTCAC-3' | 5'RACE-R1 |
| --- | --- |
| 5'-AGCATCCAGGGCATCGGTTCTTTCAC-3' | 5'RACE-R2 |
| 5'-ACATGGGGCTGAGTTTCATG-3' | RtVP1-Full-F |
| 5'-AGAGGCGCCTCCTGCCTGTGAG-3' | RtVP1-Full-R |
| 5'-GCGTCGACATGATTTCAGATCTAGTAAC-3' | RtVP1-SalI-F |
| 5'-GCGTCGACAAAGATTTTGAAGAGAAGACCATG-3' | RtVP1-SalI-R |
| 5'-ATGATTTCAGATCTAGTAACTGAGATTG-3' | RtVP1-ORF-F |
| 5'-TTAAAAGATTTTGAAGAGAAGACCACCATG-3' | RtVP1-ORF-R |
| 5'-GCTCTAGAATGATTTCAGATCTAGTAACTGAGA-3' | RtVP1-XbaI-F |
| 5'-GCGAGCTCTTAAAAGATTTTGAAGAGAAGACCA-3' | RtVP1-SacI-R |
| 5'-TTGTCACTGAAGCGGGAAGG-3' | NPTⅡ-F |
| 5'-CGGCGATACCGTAAAGCAC-3' | NPTⅡ-R |
| 5'-CTGGATTCTGGTGATGGTGTGTCT-3'； | AtActin2-F |
| 5'-GAACCACCGATCCAG ACACTGTAC-3' | AtActin2-R |
| 5'-GGGGAGGGAAATGAACAA-3' | RtVP1-SP-F |
| 5'-CCACCAAGACCATAACCAG-3' | RtVP1-SP-R |
| 5'-GGAATCCACGAGACCACCTACA-3' | RtActin-RT-F |
| 5'-GATTGATCCTCCGATCCAGACA-3' | RtActin-RT-R |
| 5'-GGGACAACGCCAAGAAATACA-3' | RtVP1-RT-F |
| 5'-GCAAAGAAGGGAGCAAATACAAG-3' | RtVP1-RT-R |
| 5'-ATGAGAGGCTAACTGTCGTCGCG-3' | AtKUP8-F |
| 5'-TCGATCCTCGCACTTTCTGGTAAC-3' | AtKUP8-R |
| 5'-CGAGACGGACAAAGAAGAGGAACC-3' | AtHAK5-F |
| 5'-CACGACCCTTCCCGACCTAATCT-3' | AtHAK5-R |
| 5'- TGAGCCTTCAGGGAACCAC-3' | AtNHX1-F |
| 5'- AAAGCCACGACCTCCAAAGA -3' | AtNHX1-R |
| 5'-TCGTTTCAGCCAAATCAGAAAGT -3' | AtSOS1-F |
| 5'-TTTGCCTTGTGCTGCTTTCC -3' | AtSOS1-R |
| 5'-AACTGCCACCTTCACAATCACT-3' | AtSOD1-F |
| 5'-ATGGACAACAACAGCCCTACC-3' | AtSOD1-R |
| 5'-GCACATACGATAGGAGTCACACA-3' | AtPOD1-F |
| 5'-GACAAGCAACACGCAAGAAC-3' | AtPOD1-R |
| 5'-TCCTGTTATCGTTCGTTTCTCA-3'; | AtCAT1-F |
| 5'-CAAAGTTCCCCTCTCTGGTGTA-3' | AtCAT1-R |
| 5'-GCGCATAGTTTCTGATGCAA-3'; | AtP5CS1-F |
| 5'-TGCAACTTCGTGATCCTCTG-3' | AtP5CS1-R |
| 5'-ATGATCTTATTTATGTTCTGC-3' | AtP5CS2-F |
| 5'-CACTATCTTCCGTCACTAT-3' | AtP5CS2-R |
| 5'-TCCAGGCATTGTCCACAGAA-3' | AtActin8-F |
| 5'-ACCTGCTCCTCCTTAGACAT-3' | AtActin8-R |
| 5'-TAGCCATTGTCGTCCCTCAGATG-3' | AtSUC2-F |
| 5'-ATGAAATCCCATAGTAGCTTTGAAGG-3' | AtSUC2-R |
| 5'-AGTTCACTGCGGATATTTTCGC-3' | AtSUS1-F |
| 5'-CCCAACAGTTTCTTTGCTTCCA-3' | AtSUS1-R |
| 5'-TGCCATGAATAATGCCGATTTC-3' | AtSUS2-F |
| 5'-TTGCCCAACATTGTTCTTGCTT-3' | AtSUS2-R |
| 5'-GACCAGACTGATGAGCATGTCG-3' | AtSUS3-F |
| 5'-TCTTCACTTTGTCGAGCCTCG-3' | AtSUS3-R |
| 5'-AAGGAATCGTTCGCAAATGG-3' | AtSUS4-F |
| 5'-TTTCAGCGGCAACATCCTC-3' | AtSUS4-R |
| 5'-GCAGTGGTAATTCCTCCGAAC-3' | AtSUS5-F |
| 5'-TCCTCTTACTGCGAACGCTACG-3' | AtSUS5-R |
| 5'-CGGAGGCCAGGTTGTTTACAT-3' | AtSUS6-F |
| 5'-AGGCTTGAATCCGAGACCTTGT-3' | AtSUS6-R |
| 5'-GGCTACTTTCTTGAGCCGGT-3' | AtUGP1-F |
| 5'-CTTCACACCGGATTTTGCCG-3' | AtUGP1-R |
| 5'-GACAGTAAAGGCAAACGCCG-3' | AtUGP2-F |
| 5'-TCACAGATCCTCTGGACCGT-3' | AtUGP2-R |
| 5'-GAACGTCCTGGGGCGC-3' | AtAHA1-F |
| 5'-GATACCCTTCACCTTTGCAAATGT-3' | AtAHA1-R |
| 5'-TTGTTGAACGTCCTGGAGCA-3' | AtAHA2-F |
| 5'-AATTCCCAGTTGGCGTAAACC-3' | AtAHA2-R |
| 5'-CCGGAACAGCCTGGAAGAAT-3' | AtAHA3-F |
| 5'-GGGACAACGTTGGCTGTTTC-3' | AtAHA3-R |
| 5'-TTAGTGGCAGAGCCTGGGAT-3' | AtAHA4-F |
| 5'-CCATGGAGCGTTCTTTGTGC-3' | AtAHA4-R |
| 5'-CGCAGGTTACTTAGTGGCGA-3' | AtCIN1-F |
| 5'-AGACGCAGATCGCTTGATGA-3' | AtCIN1-R |
| 5'-CGGTGGATCTTGGCCAGTATTGC-3' | AtCIN2-F |
| 5'-CCAGCAATCTCGGTGTAGCCGT-3' | AtCIN2-R |
| 5'-GATTACATGGGACGTATCAAGAAGCT-3' | AtPFKα1-F |
| 5'-GAGAACATCCTGGTTTCACTATCG-3' | AtPFKα1-R |
| 5'-ATGACGGTTAAGCGTTGGTC-3' | AtPFKα2-F |
| 5'-TGCGCGTTTTGTCTCAATAG-3' | AtPFKα2-R |
| 5'-GACTGTAGGTGGGACTGCTCTC-3' | AtPFKß1-F |
| 5'-AGTTCAAGAAGTAGAGTGTGGCTG-3' | AtPFKß1-R |
| 5'-GATGAAGAAGGCCAATGGAA-3' | AtPFKß2-F |
| 5'-AGCTCGGTTTCAACCATTTG-3' | AtPFKß2-R |
| 5'-GCCAATCTCAGTGGTTCGTCAA-3' | AtSWEET11-F |
| 5'-GAAGAGGACTGCTTGCCATGT-3' | AtSWEET11-R |
| 5'-CTCACATCTCCTGAACCAGTAGC-3' | AtSWEET12-F |
| 5'-TGCAGCACTGTTTCTAACTCCC-3' | AtSWEET12-R |
